# Effect of Halide Tuning on the Structural, Dielectric, and Optical Properties of Two-Dimensional 2-Chloroethylammonium Lead Halides

**DOI:** 10.1021/acs.inorgchem.4c05340

**Published:** 2025-02-26

**Authors:** Mirosław Mączka, Jan Kudrawiec, Katarzyna Fedoruk-Piskorska, Dagmara Stefańska, Anna Gągor, Marek Drozd, Szymon Smółka, Adam Sieradzki

**Affiliations:** † Institute of Low Temperature and Structure Research, Polish Academy of Sciences, Okólna 2, Wroclaw 50-422, Poland; ‡ Department of Experimental Physics, Wrocław University of Science and Technology, Wybrzeże Wyspiańskiego 27, Wrocław 50-370, Poland

## Abstract

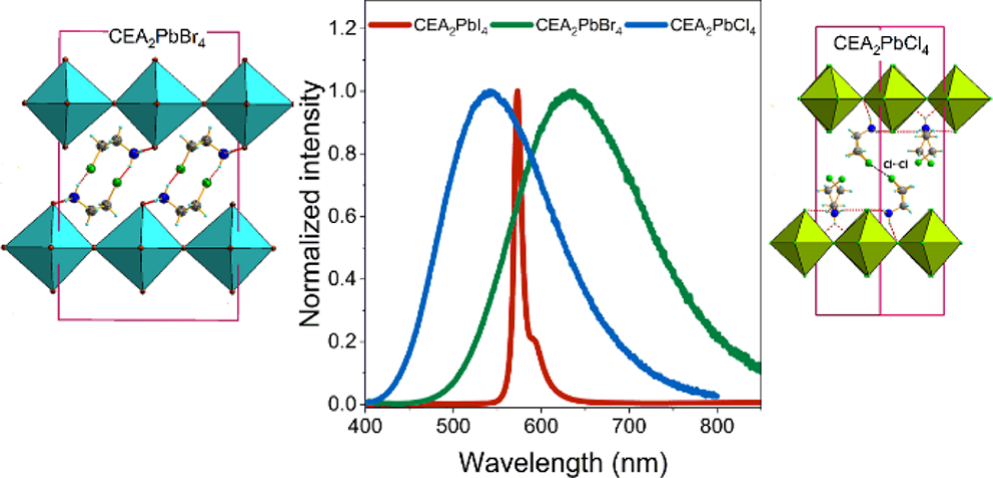

Layered hybrid organic–inorganic lead halides have gained a lot of attention for optoelectronic applications. A notable subset within this category is perovskites comprising halogenated amines since they may exhibit reduced band gap or polar order. We synthesized three compounds comprising 2-chloroethylammonium (CEA^+^) cations, with the chemical formula CEA_2_PbX_4_ (X = Cl, Br, I). X-ray diffraction studies show that at room temperature (RT), CEA_2_PbBr_4_ and CEA_2_PbI_4_ crystallize in *Pbnm* symmetry, with ordered CEA^+^ cations. CEA_2_PbBr_4_ and CEA_2_PbI_4_ undergo one structural phase transition (PT) into a disordered *Pmnm* phase near 315 and 360 K, respectively. CEA_2_PbCl_4_ shows a different packing of CEA^+^ with the organic chains oriented perpendicularly to the perovskite layers. It undergoes two PTs at 332 and 203 K from the high-temperature (HT) disordered *I*4/*mmm* phase to the partially ordered intermediate *Pbnm* phase and completely ordered low-temperature (LT) phase of the unknown space group. All compounds emit photoluminescence (PL): orange, yellow-green, and yellow for CEA_2_PbI_4_, CEA_2_PbCl_4_, and CEA_2_PbBr_4_, respectively, and bromide exhibits a very high quantum efficiency of 48%. Overall, our findings show that halide engineering strongly modulates hydrogen and halogen bonding strength, affecting the structural arrangement of building units, molecular dynamics, and thus optoelectronic properties.

## Abstract 2

Three compounds comprising 2-chloroethylammonium (CEA^+^) cations with the chemical formula CEA_2_PbX_4_ (X = Cl, Br, I) were synthesized. X-ray diffraction studies show that at room temperature (RT), CEA_2_PbBr_4_ and CEA_2_PbI_4_ crystallize in *Pbnm* symmetry, with ordered CEA^+^ cations. CEA_2_PbCl_4_ shows a different packing of CEA^+^ with the organic chains oriented perpendicularly to the perovskite layers. Overall, our findings show that halide engineering strongly modulates hydrogen and halogen bonding strength, affecting the structural arrangement of building units, molecular dynamics, and thus optoelectronic properties.

## Introduction

Three-dimensional (3D) hybrid inorganic–organic lead halide perovskites of the general formula APbX_3_, where A denotes a small organic cation and X stands for Cl, Br, and I, have emerged as potential components of new-generation solar cells, light-emitting diodes, lasers, photodetectors, nonlinear optical (NLO) devices, and dielectric switches.^1−6^

Since only a handful of small organic cations allow the synthesis of 3D perovskites,^5−12^ synthesis efforts were directed toward lower-dimensional systems, especially two-dimensional (2D) perovskites with A_2_PbX_4_ and A’PbX_4_ formulas, where A and A’ denote monovalent and divalent organic cations, respectively.^13−15^ In these compounds, corner-sharing PbX_6_ octahedra form layers separated by organic cations.^13,14,16^ What is promising in these types of structures is their higher stability at RT compared to 3D analogues.^13^ Furthermore, since the organic cations are not constrained in small perovskite voids, as in 3D analogues, using various amines allows the synthesis of almost an infinite number of such systems. Similar to 3D perovskites, 2D counterparts are also semiconductors, but their band gaps are wider and exciton binding energies larger.^13,17,18^ Due to these features, they are less attractive for photovoltaic applications compared to 3D analogues, but they are promising light-emitting materials.^13^ Note that in contrast to 3D perovskites, which exhibit narrow photoluminescence (PL) due to radiative recombination of free excitons (FE), 2D analogues may exhibit both FE-related PL as well as broadband emission, usually attributed to self-trapped excitons (STEs).^13,14,16^ Compounds exhibiting such broadband emission, including white-light emission, are widely sought since they are promising for all kinds of optic emitters and lighting.^13^ It is worth noting that many 2D lead halide perovskites undergo structural phase transitions (PTs) associated with the disordering of organic cations at elevated temperatures.^19,20^ Such structural transformations may be associated with pronounced changes in the dielectric permittivity or loss of the inversion center, leading to switchable dielectric, ferroelectric, piezoelectric, or second-order NLO properties, useful for the construction of various switches, sensors, and many other optoelectronic devices.^6,20−23^

One of the interesting series of 2D perovskite materials comprises alkylammonium and arylammonium cations (R-NH_3_^+^).^14,24,25^ Such structures are stable under ambient conditions, but some of them may exhibit structural PTs, which affect their functional properties.^26,27^ The replacement of the alkylammonium or arylammonium cations with other organic cations is an efficient way of tuning the structural diversity and properties of 2D perovskites. In this respect, the application of small cations with high dielectric permittivity, such as methylhydrazinium^20,23,28^ or ethanolammonium,^29−33^ leads to a pronounced decrease of the dielectric confinement and thus a narrowing of the band gap and decrease of the exciton binding energy, which is beneficial for photovoltaic applications. Another promising direction is the employment of halogenated ammonium cations.^34−37^ Indeed, it has been shown that lead iodides comprising 2-bromoethylammonium (BEA^+^) and 2-chloroethylammonium (CEA^+^) cations show strongly reduced band gaps.^34^ Very recently, BEA_2_PbBr_4_ was reported to exhibit ultralow ion migration and very high sensitivity for X-ray photodetection.^37^ Latter studies of iodoalkylammonium lead iodides revealed that such 2D perovskites may also exhibit interesting PL properties useful for light-emitting applications.^35^ Changing I to Cl in the 2-haloethylammonium cation and I^–^ to Cl^–^ or Br^–^ in the perovskite layers should affect the crystal structure, lattice dynamics, and optical properties (band gap and PL). However, the PL properties of CEA_2_PbI_4_ have not been reported, and the only available data are its optical absorption and RT crystal structure.^34^ Furthermore, up to now, CEA_2_PbBr_4_ and CEA_2_PbCl_4_ analogues have not been synthesized.

In this work, we report the synthesis of known CEA_2_PbI_4_ and two novel perovskites comprising CEA^+^ cations, namely CEA_2_PbBr_4_ and CEA_2_PbCl_4_. We employed a multitechnique approach (X-ray diffraction, thermal analysis, Raman, IR, absorption, PL, and dielectric spectroscopy) to obtain information on their crystal structures and dielectric, phonon, and optical properties, as well as to elucidate the mechanism of the discovered PTs. Overall, our study fills a gap in previously described crystal structures and highlights the impact of halide substitution on the optoelectronic properties and lattice dynamics of 2D perovskites comprising CEA^+^ cations.

## Experimental Section

### Synthesis

All reagents were commercially purchased from Sigma-Aldrich and used without further purification (PbI_2_ 99%, PbCl_2_ 99%, PbBr_2_ 99%, HI 57% in H_2_O, HBr 48% in H_2_O, HCl 37% in H_2_O, 2-chloroethylamine hydrochloride).

In order to grow single crystals of CEA_2_PbI_4_, 2 mmol of PbI_2_ and 5 mmol of 2-chloroethylamine hydrochloride were dissolved in HI at RT until a clear solution was obtained. The solution was kept at RT, and red crystals that grew at the bottom of the vial were separated from the liquid after 1 day and dried at RT. The same synthesis method was used for CEA_2_PbBr_4_, but for this compound, the plate-like crystals had a pale yellow color.

This procedure failed in the case of CEA_2_PbCl_4_ since due to the poor solubility of PbCl_2_, only PbCl_2_ crystals grew after a few days. We have, therefore, modified the procedure. At first, 2 mmol of PbCl_2_ were dissolved in 10 mL of HCl after stirring for 20 min on a hot plate at 60 °C. In a separate vial, 10 mmol of 2-chloroethylamine hydrochloride was dissolved in 5 mL of HCl at RT. The solutions were mixed and stirred. The clear solution was left at RT, and plate-like colorless crystals that grew in the vial were filtered off and dried at RT.

Photographs of the obtained crystals are presented in Figure S1. The experimental powder diffraction patterns of the ground crystals show good agreement with the patterns simulated based on the RT single-crystal data, confirming their purity (Figure S2).

### Powder X-Ray Diffraction

Powder X-ray diffraction (PXRD) patterns of the ground crystals were measured in the reflection mode using an X’Pert PRO X-ray diffraction system equipped with a PIXcel ultrafast line detector and Soller slits for CuKα_1_ radiation (λ = 1.54056 Å).

### Differential Scanning Calorimetry (DSC) and Thermogravimetric (TG) Measurements

DSC was performed by using a Mettler Toledo DSC-3 calorimeter in a nitrogen atmosphere. The heating/cooling rate was 5 K min^–1^, and the measured sample masses were 17.95 mg for CEA_2_PbBr_4_, 10.04 mg for CEA_2_PbCl_4_, and 13.08 mg for CEA_2_PbI_4_. The excess heat capacity associated with the PTs was calculated by subtracting from the data the baseline representing the system variation in the absence of PT.

A thermogravimetric (TG) study was performed in the temperature range of 300–1200 K using a PerkinElmer TGA 4000. The sample weights were approximately 43.63, 20.87, and 12.15 mg for the I, Br, and Cl samples, respectively. The heating speed was 10 K/min. Pure nitrogen gas was used as the atmosphere.

### Single-Crystal X-Ray Diffraction

Single-crystal X-ray diffraction data were collected using omega scans with Δω = 1° on a four-circle Xcalibur diffractometer equipped with an Atlas CCD camera and Mo Kα radiation. Absorption effects were corrected using multiscan methods in CrysAlis PRO 1.171.42.93a (Rigaku Oxford Diffraction, 2023) and spherical harmonics implemented in the SCALE3 ABSPACK scaling algorithm. Crystal structures were solved in Olex2 using SHELXT and refined with SHELXL.^38−40^ Hydrogen atoms were placed in calculated positions for the low-temperature (LT) phases of both compounds and refined as riding atoms. Crystallographic results and refinement details are given in Table 1. All CEA_2_PbX_4_ perovskites undergo symmetry reduction upon cooling from their high-temperature (HT) phases. The orthorhombic space groups were determined based on the unit cell orientation of the highest symmetry tetragonal phase, *I*4/*mmm*, with the *c*-axis perpendicular to the perovskite layers. This approach led to nonstandard space group settings for the lower-symmetry phases, which, however, are consistent with the previously reported crystal structure of CEA_2_PbI_4_.^34^ The main geometrical details concerning hydrogen bond (HB) geometry are listed in Table S1. Octahedral distortion parameters were calculated in Vesta.^41^

**Table 1:** Crystal Structure, Data Collection, and Refinement Results for CEA_2_PbX_4_

	CEA_2_PbI_4_	CEA_2_PbBr_4_		CEA_2_PbCl_4_	
***Crystal***					
Chemical formula	I_4_Pb·2(C_2_H_7_ClN)	Br_4_Pb·2(C_2_H_7_ClN)		Cl_4_Pb·2(C_2_H_7_ClN)	
Crystal system, space group	Orthorhombic, *Pmnm*	Orthorhombic, *Pmnm*	Orthorhombic, *Pbnm*	Tetragonal, *I*4/*mmm*	Orthorhombic, *Pbnm*
Temperature (K)	370	330	290	350	290
*a*, *b*, *c* (Å)	6.454 (4), 6.430 (4), 21.290 (2)	6.0738(4), 6.1966(4), 20.1901(11)	6.0779(4), 12.3351(6), 19.7276(11)	5.5218(3), 5.5218(3), 25.244(6)	7.7725(4), 7.8542(6), 24.918(3)
α, β, γ (deg)	90, 90, 90	90, 90, 90	90, 90, 90	90, 90, 90	90, 90, 90
*V* (Å^3^)	883.5(1)	759.89 (8)	1479.0 (2)	769.7 (2)	1521.2 (2)
*Z*	2	2	4	2	4
***Data collection***					
No. of meas., indep., and obs. ref [*I* > 2σ(*I*)]	2738, 1170, 927	4935, 1069, 895	4938, 1839, 1377	1095, 317, 260	8770, 1920, 1254
*R*_int_	0.026	0.039	0.040	0.049	0.074
***Refinement***					
*R*[*F*^2^ > 2σ(*F*^2^)], *wR*(*F*^2^), *S*	0.044, 0.123, 1.1	0.039, 0.091, 1.09	0.037, 0.088, 1.01	0.044, 0.103, 1.08	0.059, 0.120, 1.11
No. of reflections	1170	1069	1839	317	1920
No. of parameters	52	47	66	7	84
Δρ_max_, Δρ_min_ (e Å^–3^)	2.43, −1.44	1.21, −2.20	1.23, −1.95	0.99, −0.82	1.82, −2.66
σ (deg.^2^), Δ	1.2, 0.005	0.9, 0.011	7.5, 0.039	-	19.4, 0.006

### Raman Spectroscopy

RT Raman spectra were measured for powdered crystals using a Bruker FT MultiRam spectrometer with YAG:Nd laser excitation (λ_exc_ = 1064 nm) and 2 cm^–1^ spectral resolution. The same instrument was used for recording polarized Raman spectra of CEA_2_PbBr_4_ at RT (polarized spectra were not measured for iodide and chloride due to the too small size and thickness of the crystals). Temperature-dependent Raman spectra were measured using a Renishaw inVia Raman spectrometer equipped with a confocal DM2500 Leica optical microscope and a CCD detector. For CEA_2_PbBr_4_, the excitation was done using an argon laser (λ_exc_ = 514 nm), but due to the luminescence background, Raman spectra of CEA_2_PbCl_4_ and CEA_2_PbI_4_ were measured using a diode laser (λ_exc_ = 830 nm). The temperature of the samples was controlled by using a THMS600 temperature control stage (Linkam). The spectral resolution of all Raman spectra was 2 cm^–1^.

### IR Spectroscopy

RT IR spectra of all samples were recorded by using a Nicolet iS50 FT-IR spectrometer. Both attenuated total reflection (ATR) and the standard KBr pellet method were employed. Temperature-dependent IR spectra were recorded for CEA_2_PbCl_4_ only in the 10–300 K range using a Nicolet iS50 FT-IR spectrometer and a closed cycle cryostat CS202AE-DMX-1AL (Advanced Research Systems) equipped with thallium bromoiodide (KRS-5) windows. Since this compound reacted with KBr, the sample for the temperature-dependent studies was diluted in Apiezon. The spectral resolution of all of the IR spectra was 2 cm^–1^.

### Broadband Dielectric Spectroscopy (BDS)

Broadband dielectric spectroscopy was carried out by using a Broadband Impedance Novocontrol Alpha-A analyzer. Since the obtained single crystals were not large enough to perform single-crystal dielectric measurements, pellets were measured. The powder was pressed into cylindrical pellets 5 mm in diameter and about 0.5–0.3 mm in thickness. Silver paste was deposited on the pellet surface to ensure good electrical contact. A sinusoidal voltage with an amplitude of 1 V and a frequency in the range of 1 Hz–1 MHz was applied across the samples. The temperature was stabilized using nitrogen gas with the Novocontrol Quattro system.

### Optical Absorption and Photoluminescence (PL) Studies

The RT diffuse reflectance spectra of the powdered samples were measured by using the Varian Cary 5E UV–vis-NIR spectrophotometer. PL spectra as a function of temperature were measured using a Hamamatsu photonic multichannel analyzer PMA-12 equipped with a BT-CCD linear image sensor. Laser diodes of 266, 375, and 450 nm were used as the excitation source for CEA_2_PbCl_4_, CEA_2_PbBr_4_, and CEA_2_PbI_4_, respectively. The temperature of the samples during emission measurements was controlled by applying a Linkam THMS 600 Heating/Freezing Stage. A femtosecond laser (Coherent Model Libra; Coherent, Pennsylvania, USA) was used as an excitation source to record decay times. The PL quantum yield (PLQY) of the CEA_2_PbBr_4_ sample was measured at 80 K under a 360 nm excitation line using the Fluorescence Quantum Yields with an FLS920 spectrometer. Due to equipment limitations (for CEA_2_PbCl_4_) and low emission intensity (for CEA_2_PbI_4_), PLQY measurements were not possible for the remaining samples.

## Results and Discussion

### TG and DSC

The TG plots indicate that CEA_2_PbX_4_ decomposes at ∼528, 520, and 493 K for the I, Br, and Cl analogues, respectively (Figures S3–S5). In these plots, a weight loss of ∼43.9, 41.1, and 45.8% takes place between 528 and 645, 520–672, and 493–648 K for the I, Br, and Cl analogues, respectively, corresponding to the release of 2-chloroethylammonium halides (the calculated values are 47.4, 45.5, and 43.9%, respectively). On further heating, PbX_2_ starts to sublime at ∼770, ∼800, and ∼790 K for the I, Br, and Cl compounds, and this process ends above 900 K (Figures S3–S5).

**Figure 1:**
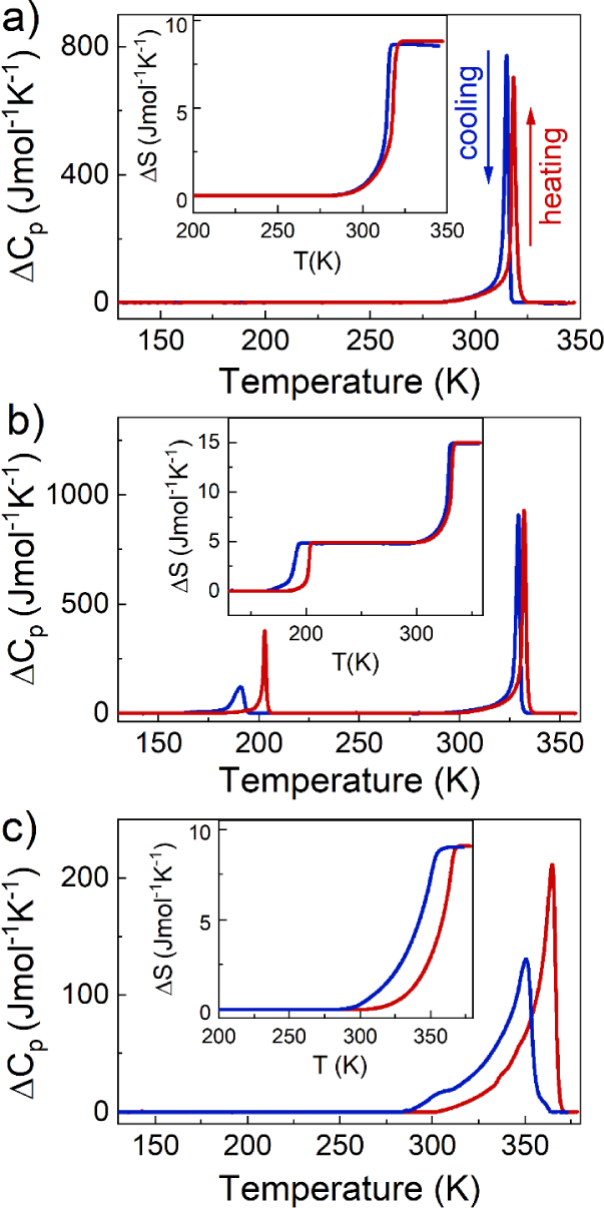
Changes in heat capacity (ΔC_p_) and entropy (Δ*S*) as a function of temperature for (a) CEA_2_PbBr_4_, (b) CEA_2_PbCl_4_, and (c) CEA_2_PbI_4_.

The calorimetric measurements show one reversible PT at T_1_ = 315.7 (313.2 K) for CEA_2_PbBr_4_ (Figure S6a), two PTs at T_1_ = 332.1 (329.2 K) and T_2_ = 203.0 (190.9 K) for CEA_2_PbCl_4_ (Figure S6b), and one PT at T_1_ = 364.7 (350.7 K) for CEA_2_PbI_4_ (Figure S6c) during heating (cooling) modes. The symmetrical peaks seen in ΔC_p_ as a function of temperature, in combination with the thermal hysteresis between heating and cooling cycles, indicate the first-order type of all PTs (Figure 1). This is also confirmed by the discontinuous change in the entropy (Δ*S*) (see the inset in Figure 1). The change in entropy (Δ*S*) was estimated to be 8.9 J·mol^–1^·K^–1^ for CEA_2_PbBr_4_ and 9.0 J·mol^–1^·K^–1^ for CEA_2_PbI_4_. The associated changes in entropy Δ*S* for CEA_2_PbCl_4_ were estimated to be 10 and 4.9 J·mol^–1^·K^–1^ for the PT at T_1_ and T_2_, respectively. Based on the Boltzmann equation, Δ*S* = Rln(N), where Δ*S* is the average entropy change, R is the gas constant, and N denotes the ratio of the number of distinguishable states. The estimated N is approximately 3 for PTs at T_1_ for all samples, while in the case of CEA_2_PbCl_4_ at T_2_, this value is around 2. The large values of N point to a significant contribution of an order–disorder mechanism to the PTs.

### Single-Crystal X-Ray Diffraction

The crystal structures of CEA_2_PbX_4_ are built of layers of corner-sharing PbX_6_ octahedra, representing [100] cuts of the perovskite structure. The CEA^+^ cations are arranged in bilayers between the perovskite sheets. In layered organic–inorganic compounds, octahedral distortions and tilting of octahedra in the equatorial PbI_2_ planes play a key role in determining their electronic structure.^42,43^ These structural alterations are primarily driven by weak noncovalent forces at the organic–inorganic interfaces. Among these, the HBs between ammonium groups and halide ions, as well as halogen–halogen interactions, are the most significant.^34,44−46^ The example of CEA_2_PbX_4_ demonstrates how halide engineering can modulate these interactions. By influencing both the strength of noncovalent forces and altering geometric factors, halide modifications affect the symmetry and structural arrangements within the inorganic layers.

The crystal structure of CEA_2_PbBr_4_ is isomorphic at RT to that of CEA_2_PbI_4_, both exhibiting an orthorhombic symmetry of the *Pbnm* space group.^34^ The interlayer distances in both compounds are similar, measuring half of the parameter *c*, specifically 10.40 Å for CEA_2_PbI_4_ and 10.09 Å for CEA_2_PbBr_4_. In both compounds, the CEA^+^ cations are oriented parallel to the perovskite layers, with NH_3_ groups involved in two N–H···X HBs with apical, nonbridging halides of the PbX_6_ octahedra. Additionally, intermolecular N–H···Cl HBs occur between adjacent CEA^+^. These interactions induce slight distortions in the inorganic framework, which are more pronounced in the bromide crystals due to the higher electronegativity of the bromide ion and the enhanced strength of the N–H···Br HBs.

Quantitatively, the octahedral distortion for bond lengths is measured at 0.039 Å for bromine compared to 0.006 Å for iodine, while the angle variance is 7.5 vs 2.5 deg^2^, respectively. The octahedral tilting in both compounds is negligible. Although the Pb–Br–Pb angles are 177° along the *a*-axis and 175° along the *b*-axis in CEA_2_PbBr_4_, and the Pb–I–Pb angles are 178° along the *a*-axis and 177° along the *b*-axis in CEA_2_PbI_4_, these changes are associated with octahedral distortions rather than rotations of octahedra.

**Figure 2:**
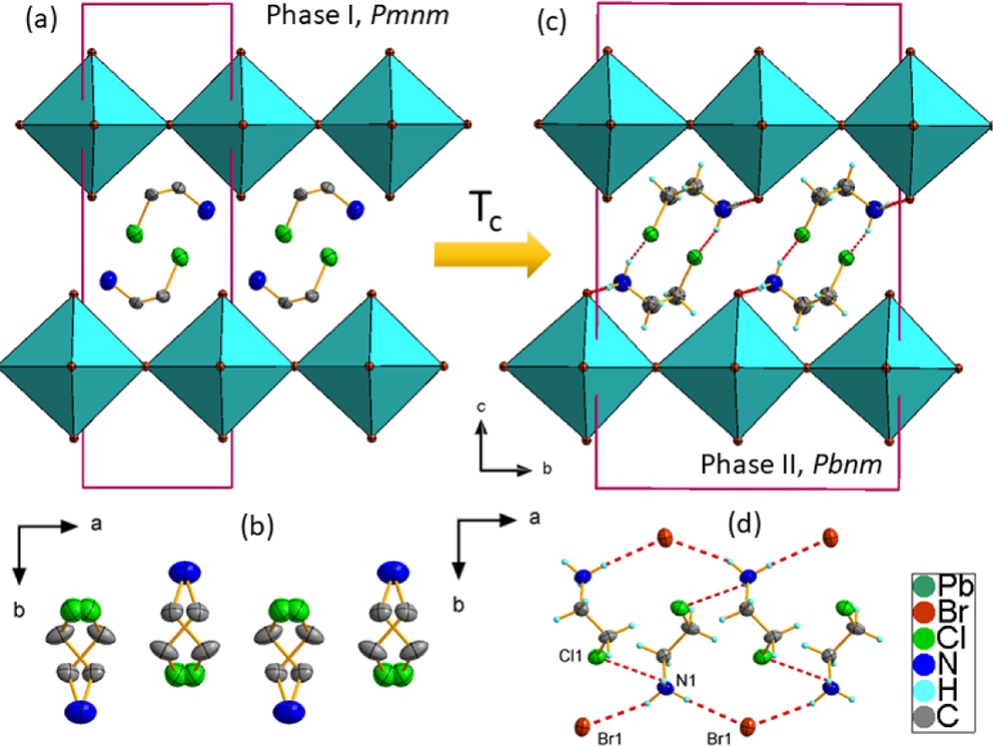
Details of the (a) disordered HT orthorhombic crystal structure of CEA_2_PbBr_4_, space group *Pmnm* at *T* = 330 K; (b) disordered CEA^+^ cations adopting two equivalent positions in the HT phase; (c) orthorhombic LT *2b* superstructure, space group *Pbnm* at *T* = 290 K, with HBs shown as dashed red lines; and (d) geometry of the HBs in detail.

Both compounds (CEA_2_PbBr_4_ and CEA_2_PbI_4_) undergo a PT to form an HT polymorph (I) with *Pmnm* symmetry and a reduced *b* lattice parameter. The RT phase, polymorph II, is a superstructure of the HT phase. The most significant structural changes during this transformation are associated with the organic moiety, which experiences thermally induced disorder. Each molecule in phase I occupies two symmetrically equivalent positions induced by the .m. mirror plane. The intermolecular N–H···Br and N–H···I HBs present in II are broken in the HT phase I. The reorganization of molecular components also leads to the weakening of the N–H···Br and N–H···I interactions at the organic–inorganic interface. Figure 2 shows the details of the disordered HT phase I and ordered LT superstructure, phase II, of CEA_2_PbBr_4_. Note that despite a two-state disorder model for the CEA^+^ cations, the DSC data suggest a 3-fold disorder. This discrepancy may indicate either more pronounced rotations of CEA^+^ cations along molecular axes in phase I or the contribution of the bromide or iodide to the PT entropy.

Substituting chlorine ions in the inorganic substructure results in a significantly different packing of CEA^+^ within the interlayer regions of the perovskite compared to what is observed in both CEA_2_PbBr_4_ and CEA_2_PbI_4_. Specifically, in CEA_2_PbCl_4_, the chains are oriented along the *c*-axis, positioned perpendicularly to the perovskite layers, with NH_3_ groups directed toward the perovskite sheets. This orientation increases the interlayer distance, thereby reducing the stability of the crystal structure and leading to two PTs and three polymorphic phases with temperature lowering. Notably, the distance between the perovskite sheets increases to 12.6 Å, compared to 10.40 Å in CEA_2_PbI_4_.

**Figure 3:**
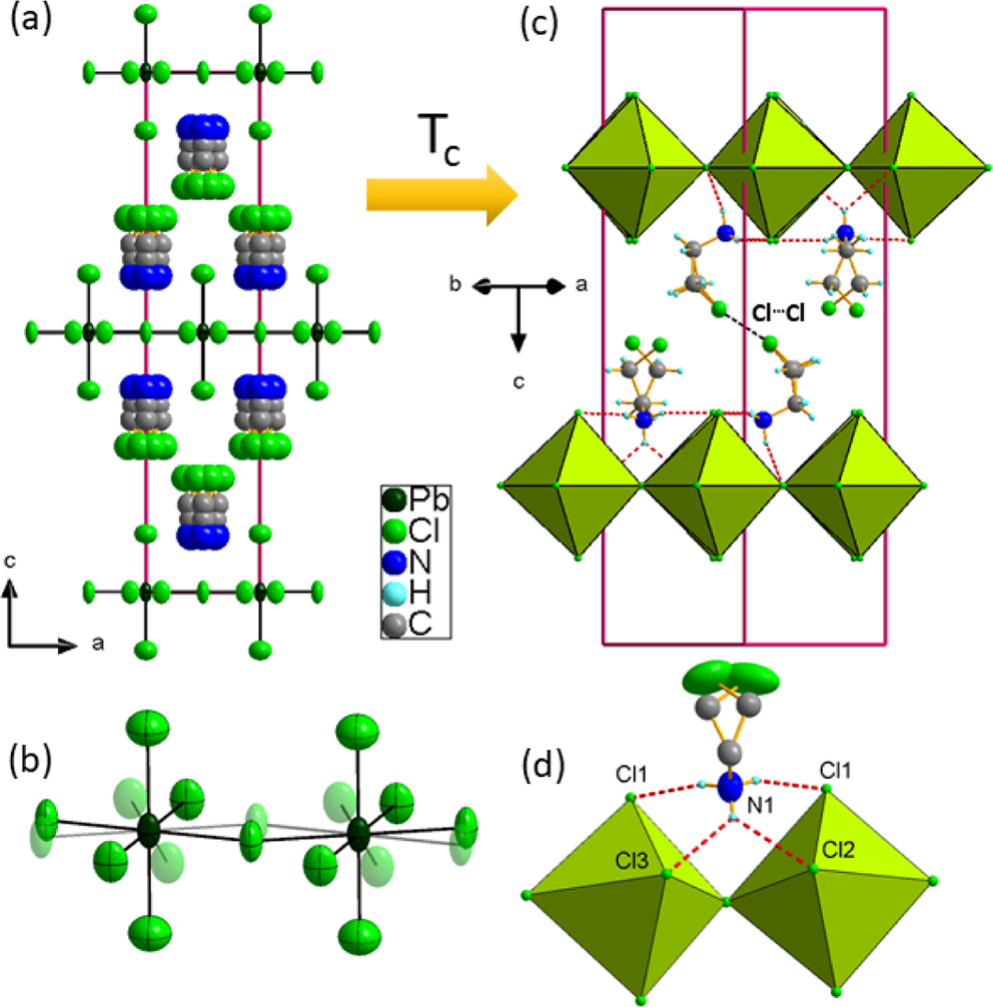
Details of the (a) disordered HT tetragonal crystal structure of CEA_2_PbCl_4_, space group *I*4/*mmm* at *T* = 350 K; (b) disordered PbCl_6_ octahedra in the HT phase I; (c) orthorhombic intermediate phase II, space group *Pbnm* at *T* = 290 K, with HBs shown as dashed red lines; and (d) geometry of the HBs in detail.

**Figure 4:**
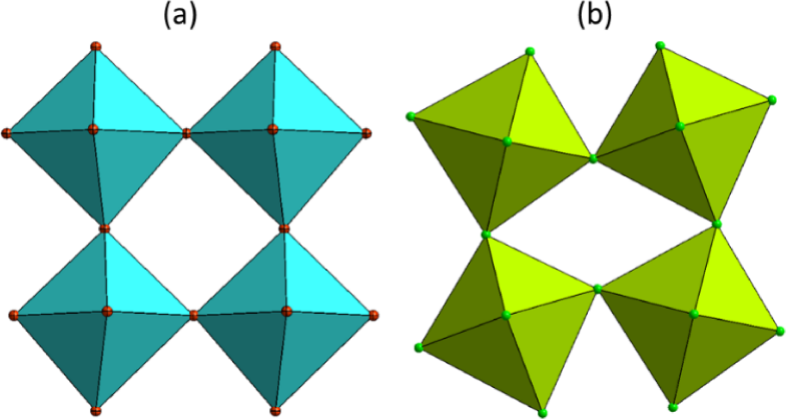
Tilting of octahedra in the RT phases of (a) CEA_2_PbBr_4_ and (b) CEA_2_PbCl_4_.

Similar to the bromide and iodide analogues, these crystals exhibit an HT disordered phase I; however, the degree of disorder is much greater, affecting both the inorganic and organic substructures. In phase I, the symmetry of the structure is tetrahedral, characterized by the *I*4*/mmm* space group. Each CEA^+^ is disordered along the 4-fold axis, with the NH_3_–C_2_H_5_ groups occupying four positions, while the chlorine atoms are additionally split across eight different sites. Meanwhile, bridging chlorine ions may occupy two equivalent sites, introducing pronounced disorder within the inorganic substructure. Figure 3a,b illustrates the main structural features of this phase. Lowering the temperature results in a symmetry reduction to orthorhombic *Pbnm*, where the [100] and [010] directions correspond to the [110] and [1–10] directions in CEA_2_PbBr_4_. RT phase II exhibits ordered inorganic layers and a significantly better-ordered organic part with only −CH_2_–Cl chains split over two nonequivalent positions with occupancies of 0.6 and 0.4. Thus, the number of distinguishable states changes from six in phase I to two in phase II, in agreement with the DSC data, which showed N ∼ 3. Importantly, the NH_3_ groups are anchored by N–H···Cl HBs exclusively with the perovskite layer. In phase II, two bonds form with nonbridging apical chlorines, and one bifurcated bond connects to two bridging chlorine ions, with donor–acceptor distances ranging from 3.228(9) to 3.40(1) Å and angles from 130° to 166°. These interactions induce strong octahedral rotations within the (001) plane. The in-plane octahedral tilting, shown in Figure 4, is significantly larger in CEA_2_PbCl_4_ compared to that in CEA_2_PbBr_4_, with Pb–Cl–Pb bond angles of 155° along [110] and 143° along [1–10]. The angle variance (σ) is 19 deg^2^, the highest among CEA_2_PbX_4_ compounds, while the bond length distortion is minimal at 0.006. The Pb–Cl distance to bridging chlorines varies between 2.85 and 2.92 Å, whereas to apical (nonbridging) chlorines, it is equal to 2.86 Å.

In CEA_2_PbCl_4_ crystals, the intermolecular HBs that stabilize the structure in the (001) planes in CEA_2_PbBr_4_ and CEA_2_PbI_4_ are absent. Instead, the geometrical parameters, such Cl···Cl distances of 3.29 Å between the molecular chlorine ions and C–Cl···Cl angles of 165°, suggest the emergence of halogen interactions between the chlorine atoms of the organic cations. In phase II, these interactions (illustrated in Figure 3c) are too weak to overcome the thermally induced dynamics of the −CH_2_–Cl groups, which remain disordered. However, they likely activate in phase III LT alongside the ordering of the CEA chains. Due to complex twinning in phase III, we were unable to model the crystal structure. However, the splitting and blurring of diffraction peaks in reciprocal space reconstructions (Figure S7) indicate significant symmetry reduction and internal strain related to the crystal structure distortion. Since phase II exhibits 2-fold disorder and the DSC data show that the change in the number of distinguishable states N is approximately two, the LT phase III is expected to be well-ordered.

### Raman and IR Study

RT Raman and IR spectra of powdered samples are presented in Figures S8 and S9, respectively. IR spectra of CEA_2_PbBr_4_ and CEA_2_PbI_4_ measured in KBr and using the ATR method show the same bands, indicating that these compounds do not react with KBr. In the case of CEA_2_PbCl_4_, the spectrum recorded in KBr differs from that measured by the ATR method but is very similar to the spectrum of CEA_2_PbBr_4_ (Figure S9). This behavior indicates that Cl^–^ is replaced by Br^–^ in the KBr pellet. Therefore, we compare the IR spectra of these compounds using ATR data only. To obtain some information on Raman modes’ symmetries, we also recorded polarized Raman spectra of CEA_2_PbBr_4_ (Figure S10). The observed modes are listed in Tables S2 and S3 together with assignments proposed based on studies of chloroethane and our previous studies of 3-chloropropylammonium lead chloride.^36,46^

RT Raman and IR spectra of CEA_2_PbBr_4_ and CEA_2_PbI_4_ are very similar (Figures S8 and S9), indicating that these compounds are isostructural. One can note that many bands are shifted to higher wavenumbers when larger I^–^ is replaced by smaller Br^–^. This type of behavior has been reported for many lead halides.^11,47^ Polarized spectra of CEA_2_PbBr_4_ show that almost all bands are seen in all polarization configurations but with different relative intensities (Figure S10). This behavior is consistent with the crystal structure, i.e., since the primitive cell contains four formula units (eight CEA^+^ cations) and all atoms are located at *C*_1_ symmetry sites, each vibration should split in the crystal into eight components, A_g_ + B_1g_ + B_2g_ + B_3g_ + A_u_ + B_1u_ + B_2u_ + B_3u_, and all gerade modes should appear in the Raman spectra. A closer inspection of the spectra shows that wavenumbers of the Raman modes exhibit weak changes depending on polarization configuration (Table S3). For instance, ν_s_(CH_2_) is observed at 2923 cm^–1^ for the A_g_ modes and at 2920 cm^–1^ for the B_1g_ mode (Table S3). This difference reflects the expected Davydov splitting, but it is small and does not exceed 5 cm^–1^.

**Figure 5:**
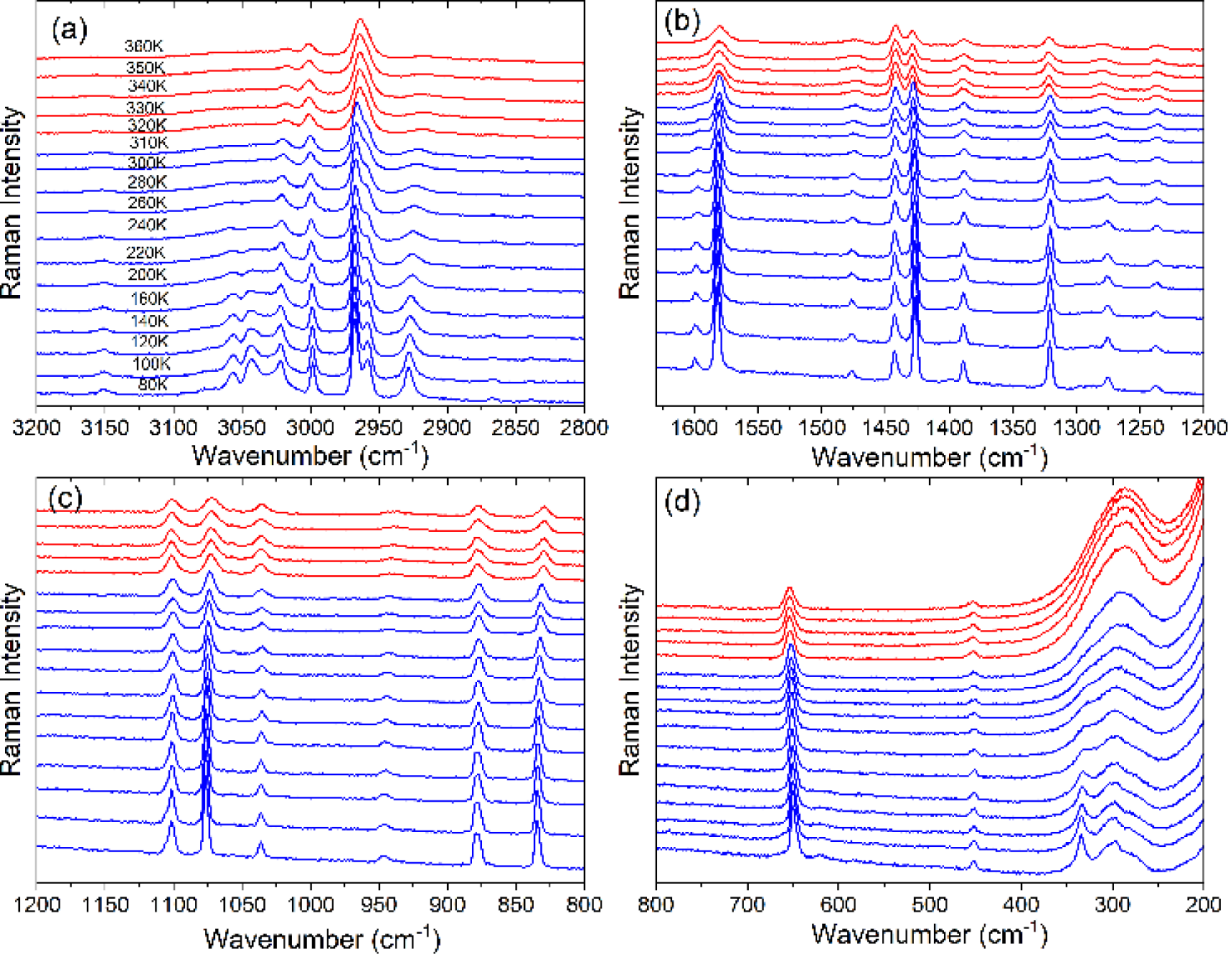
Raman spectra of CEA_2_PbBr_4_ as a function of temperature in the (a) 3200–2800 cm^–1^, (b) 1630–1200 cm^–1^, (c) 1200–800 cm^–1^, and (d) 800–200 cm^–1^ ranges. The red and blue colours correspond to the HT (I) and LT (II) phases, respectively.

Raman and IR spectra of CEA_2_PbCl_4_ differ from those of the bromide and iodide counterparts (Figures S8 and S9). For instance, the highest wavenumber IR band, corresponding to the ν_as_(NH_3_) mode, which is observed at 3167 cm^–1^ for CEA_2_PbI_4_ and shifts to 3194 cm^–1^ for CEA_2_PbBr_4_, moves to 3167 cm^–1^ for the chloride (Table S2). A large difference is also observed for the lattice modes below 200 cm^–1^ as well as for the δ_s_(NH_3_) mode, which is observed at 1472 cm^–1^ (1466 cm^–1^) in the Raman (IR) spectrum for the iodide, 1474 cm^–1^ (1473 cm^–1^) for the bromide, but at 1501 cm^–1^ (1496 cm^–1^) for the chloride (Figures 5, S8, S9 and Table S2). It is also clear that the relative intensities of bands exhibit a large change when going from the I and Br to the Cl analogues (see, for instance, the IR bands in the 3200–2900 cm^–1^ range, Figure S9). Since the most pronounced changes are observed for the N–H modes, Raman and IR data indicate that CEA_2_PbCl_4_ has a significantly different HB network than the bromide and iodide analogues.

**Figure 6:**
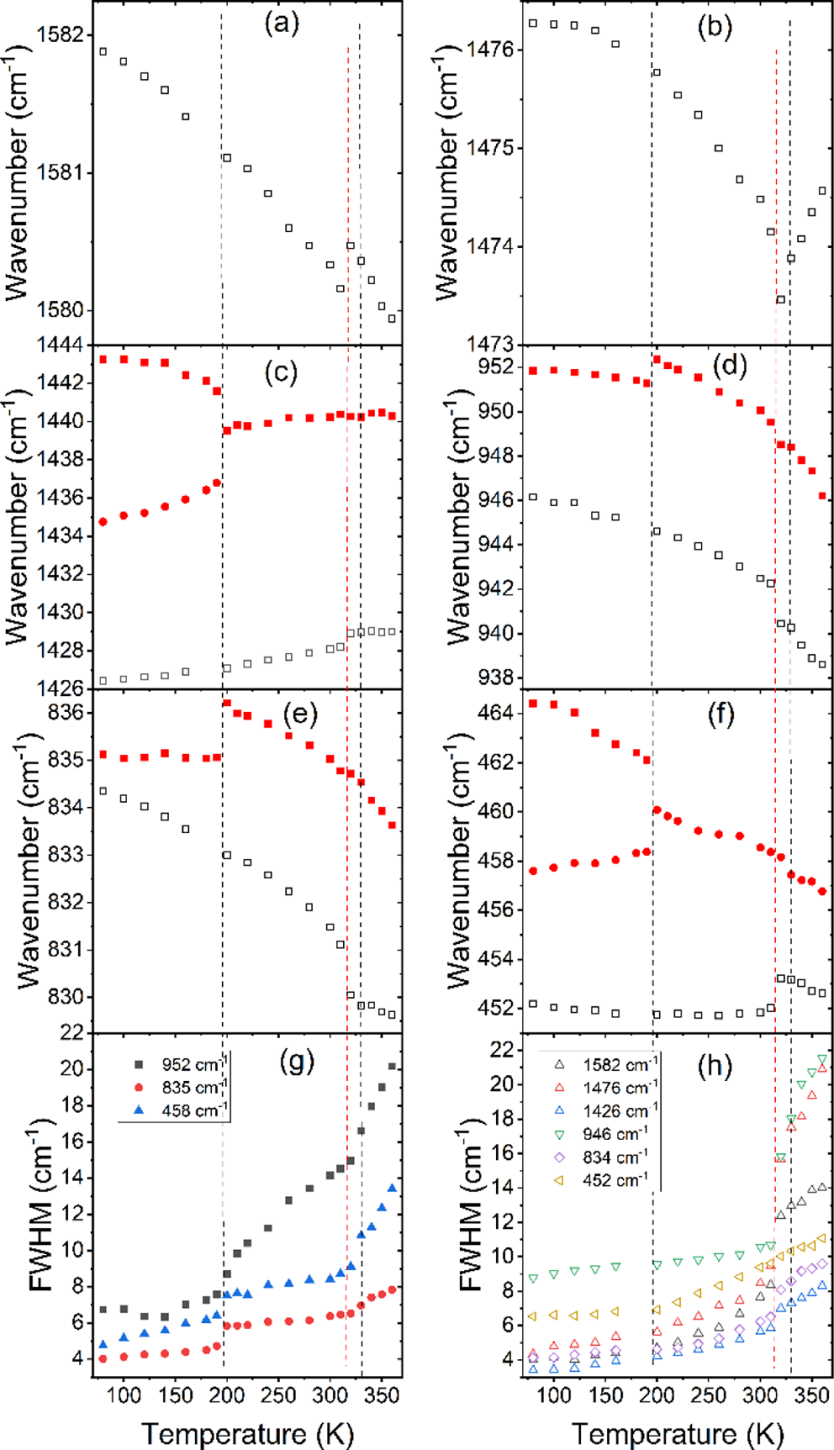
Temperature dependence of wavenumbers (a–f) and fwhm values (g,h) for CEA_2_PbBr_4_ (empty symbols) and CEA_2_PbCl_4_ (solid symbols). Dashed red (black) vertical lines correspond to the PT temperatures of CEA_2_PbBr_4_ (CEA_2_PbCl_4_).

The Raman spectrum of CEA_2_PbBr_4_ measured at 360 K shows the presence of many broad bands (Figures 5, 6h, S11). This behavior is consistent with the dynamic disorder of CEA^+^ cations in the HT phase I. When the temperature decreases, sudden shifts become evident at 310 K for many Raman bands (Figure 6a–f). Furthermore, a significant decrease in full width at half-maximum (fwhm) occurs, especially pronounced for modes related to the NH_3_ group (for instance, the δ_as_(NH_3_), δ_s_(NH_3_), and ρ(NH_3_) modes near 1582, 1476, and 946 cm^–1^, Figure 6h). The Raman data prove, therefore, that the PT in CEA_2_PbBr_4_ is associated with the ordering of CEA^+^ cations in the LT phase II and a change in the amine framework interactions. On further decrease of temperature, Raman bands exhibit usual hardening and weak narrowing (Figures 5, 6a–f,h, S11) due to lattice contraction and decrease of the phonon–phonon anharmonic interactions. The number of observed modes remains almost the same in the HT and LT phases (Table S4), indicating that both phases have the same number of unique CEA^+^ cations. Very similar behavior is also observed for CEA_2_PbI_4_, i.e., very large broadening of Raman bands in the HT phase at 390 K and pronounced narrowing of many bands on cooling, especially the δ_as_(NH_3_) and δ_s_(NH_3_) ones near 1575 and 1471 cm^–1^ (Figure S12) as well as a small change in the number of bands when going from the HT to the LT phase (Table S5). This behavior is in agreement with the same crystal structure of the iodide and bromide.

**Figure 7:**
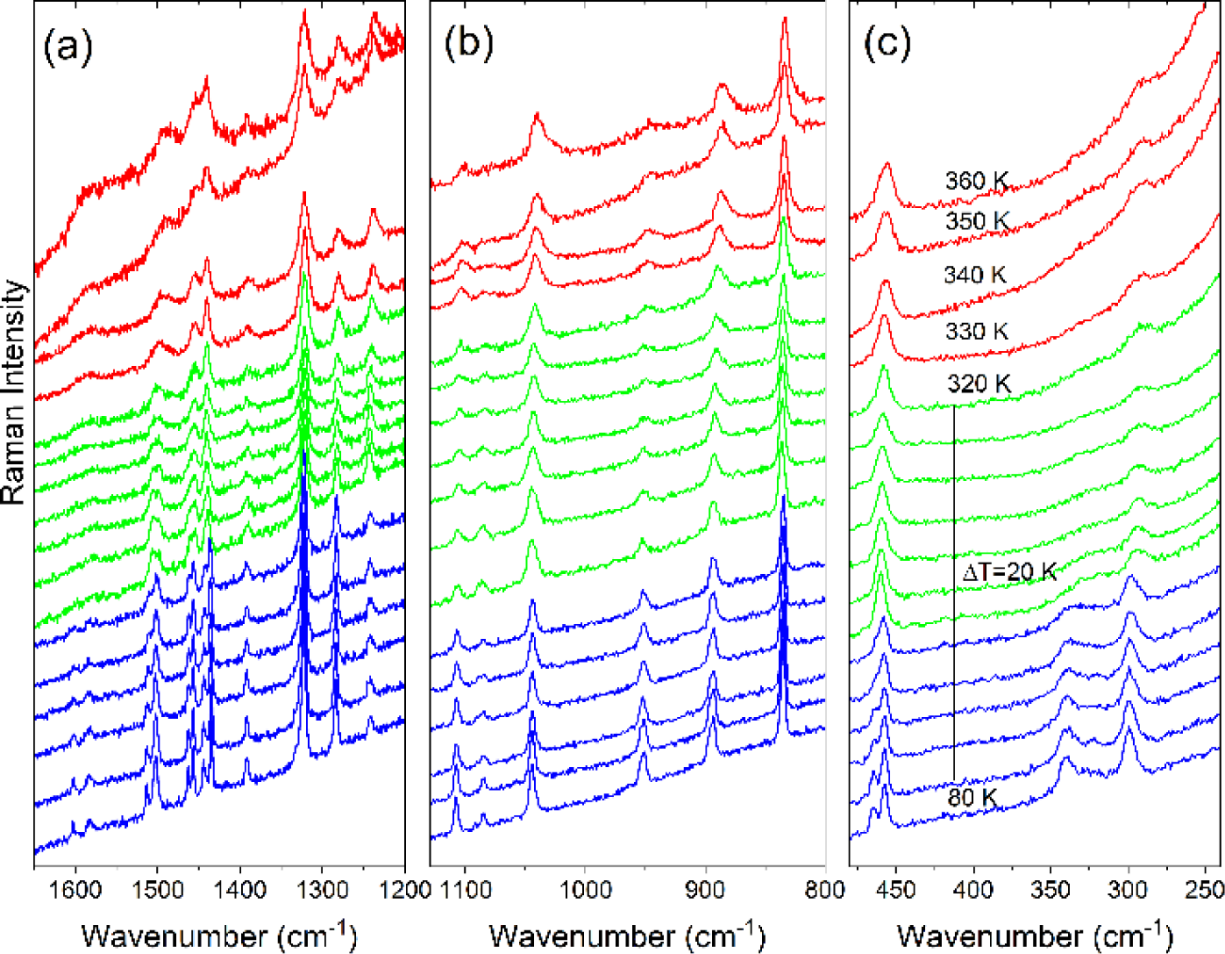
The Raman spectra of CEA_2_PbCl_4_ as a function of temperature in the (a) 1650–1200 cm^–1^, (b) 1130–800 cm^–1^, and (c) 480–240 cm^–1^ range. Red, green, and blue colors correspond to the HT (I), intermediate (II), and LT (III) phases, respectively.

**Figure 8:**
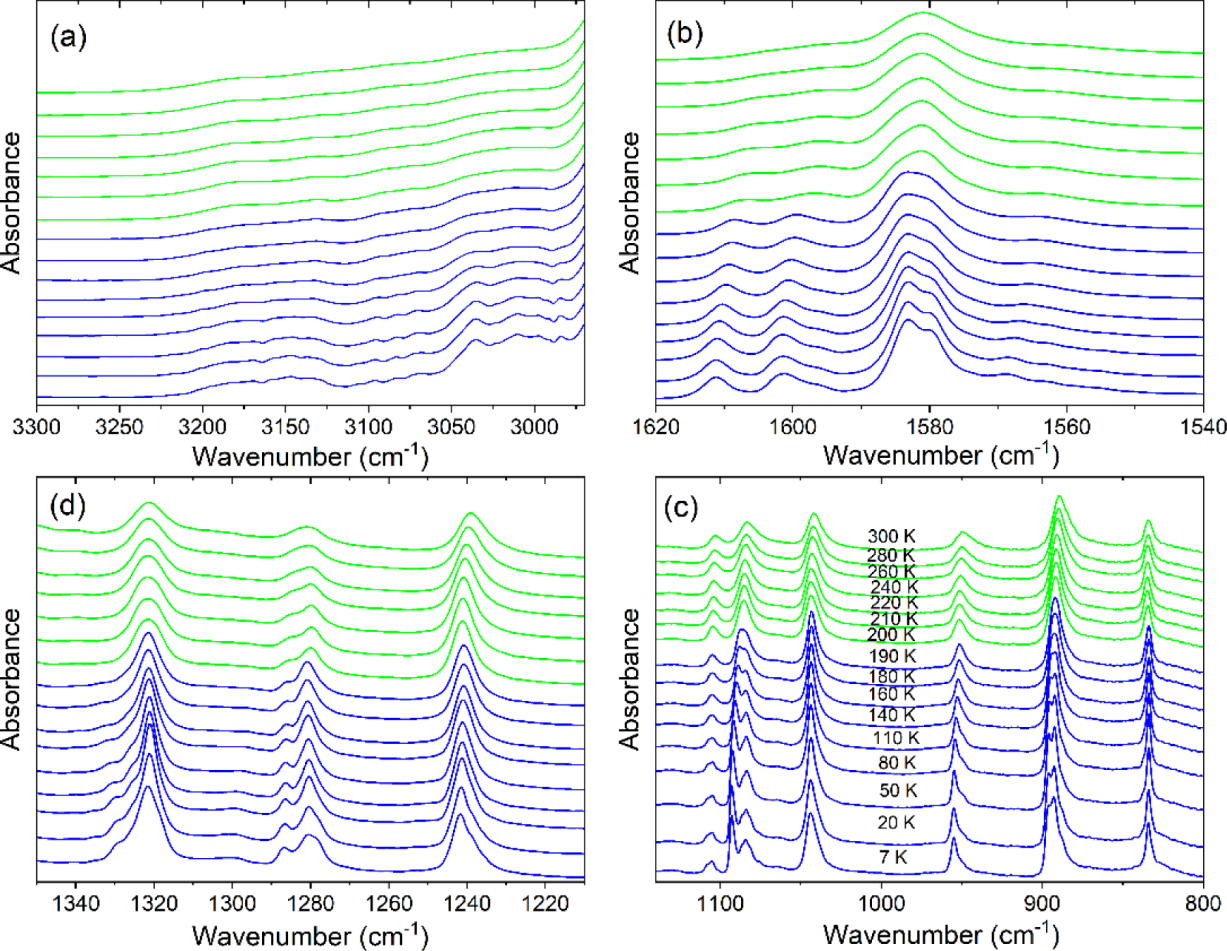
The IR spectra of CEA_2_PbCl_4_ as a function of temperature in the (a) 3300–2970 cm^–1^, (b) 1620–1540 cm^–1^, (c) 1350–1210 cm^-1^, and (d) 1140–800 cm^–1^ range. Green and blue colors correspond to the intermediate (II) and LT (III) phases, respectively.

The Raman spectrum of CEA_2_PbCl_4_ recorded at 360 K also shows relatively broad bands, indicative of pronounced disorder in the HT phase I (Figures 7, S13). On temperature lowering, no clear changes in the Raman spectra are observed, which could indicate the onset of the PT near 330 K. A closer inspection shows, however, that the fwhm of some bands exhibits a change of slope near 330 K (Figure 6g). This behavior indicates that the PT at T_1_ is related to some change in the dynamics of organic cations, but the intermediate phase II remains disordered. On further decrease of temperature, both Raman and IR modes show continuous narrowing and shifts down to 210–200 K, where the spectra exhibit sudden shifts and changes in the relative intensities of the bands. This behavior is clear for both the internal modes of CEA^+^ (Figures 6d,e, 7, 8, S13a, S14) and lattice modes (S13b). Furthermore, many bands split into doublets (Figures 6c,f, 7, 8, and S13, S14, Tables S6, S7) or show a weak but clear decrease in fwhm values (Figure 6g). The observed narrowing of bands and very small fwhm of Raman bands in the LT phase indicate that the PT at T_2_ leads to the complete ordering of CEA^+^ cations, in agreement with X-ray diffraction and DSC data. The ordering leads to significant strengthening of HBs, which in turn leads to increased deformation of the inorganic subnetwork and symmetry decrease. The splitting of many bands into doublets indicates that the number of unique CEA^+^ cations doubles in the LT phase III.

### Dielectric Properties

**Figure 9:**
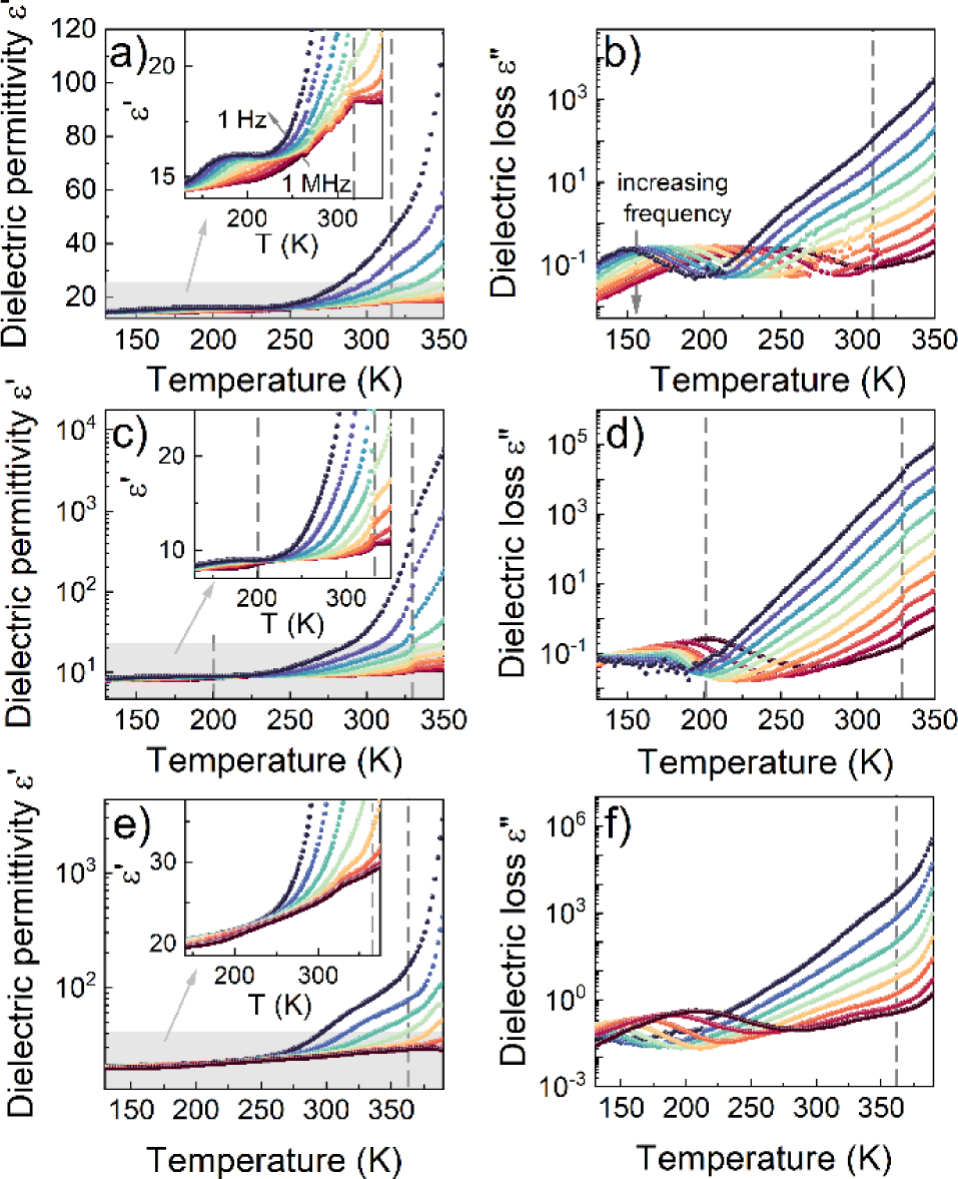
Temperature dependence of real and imaginary parts of dielectric permittivity for (a,b) CEA_2_PbBr_4_, (c,d) CEA_2_PbCl_4_, and (e,f) CEA_2_PbI_4_.

Broadband dielectric spectroscopy experiments were conducted to elucidate the structural dynamics of the compounds across all phases. Figure 9 illustrates the temperature dependence of the real (ε’) and imaginary (ε″) parts of the complex dielectric permittivity measured between 130 and 350 K. Two characteristic behaviors can be generalized as a function of temperature increase in all cases. The first occurs at the lowest temperatures, where the complex dielectric permittivity shows relatively small values for probe frequencies ranging from 1 Hz to 1 MHz. This behavior is characterized by a step-like change in ε’ and a single bell-shaped loss peak in ε″, which progressively shifts toward higher frequencies with increasing temperature. The second phenomenon, occurring above 250 K, is a significant increase in the values of both ε’ and ε″, indicating the occurrence of the ionic conduction process. Interestingly, the changes at the PT temperatures are subtle for the CEA_2_PbBr_4_ and CEA_2_PbCl_4_ compounds. Nevertheless, in the case of CEA_2_PbBr_4_, the PT is clearly observed at 1 MHz since ε’ rises by about 3.7 from 200 to 320 K and shows no increase above T_1_. CEA_2_PbCl_4_ shows two clear steps in ε’ at 1 MHz: the first is observed near 200 K with a 0.7 change, and the second larger step of 1.2 is observed near 330 K. The presence of step-like anomalies confirms the unlocking of CEA^+^ motions at the PTs. However, CEA_2_PbI_4_ does not show any significant changes at the PT temperature.

**Figure 10:**
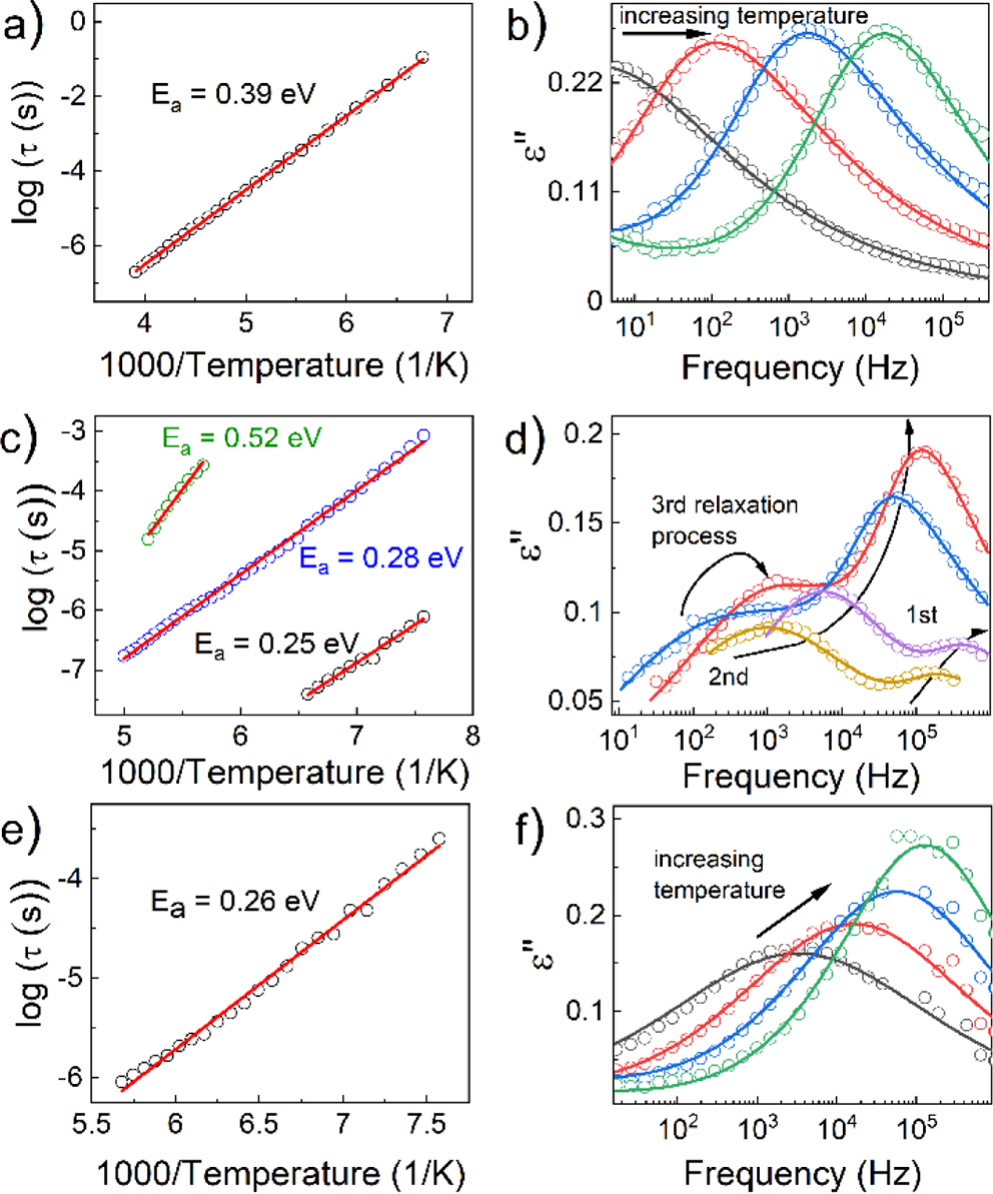
Inverse temperature dependence of the relaxation time for (a) CEA_2_PbBr_4_, (c) CEA_2_PbCl_4_, and (e) CEA_2_PbI_4_. Examples of fitting dielectric loss ε″ curves using the Havriliak–Negami function for (b) CEA_2_PbBr_4_, (d) CEA_2_PbCl_4_, and (f) CEA_2_PbI_4_.

More in-depth frequency domain analysis revealed the dipolar relaxation processes in CEA_2_PbBr_4_ (Figure S15 a,b), CEA_2_PbCl_4_ (Figure S15 c,d), and CEA_2_PbI_4_ (Figure S13 e,f). For the bromide and iodide samples, only one relaxation process is observed, which can be described well with a single Havriliak–Negami (HN) fitting function (Figure 10b,f). However, the structural dynamics of the chlorine-containing sample are more complex, exhibiting three distinct relaxation processes, which require the HN functions. All observed dielectric relaxation processes are likely attributed to some residual motions of the CEA^+^ cation, known as persistent disorder.^48^ As depicted in Figure 10a,c,e, the relaxation times of all observed relaxation processes are a linear function and parametrized using the Arrhenius equation with the activation energies *E*_a_ equal to 0.39 eV for CEA_2_PbBr_4_, 0.26 eV for CEA_2_PbI_4_, as well as 0.25, 0.28, and 0.52 eV for CEA_2_PbCl_4_.

### Optical Properties

**Figure 11:**
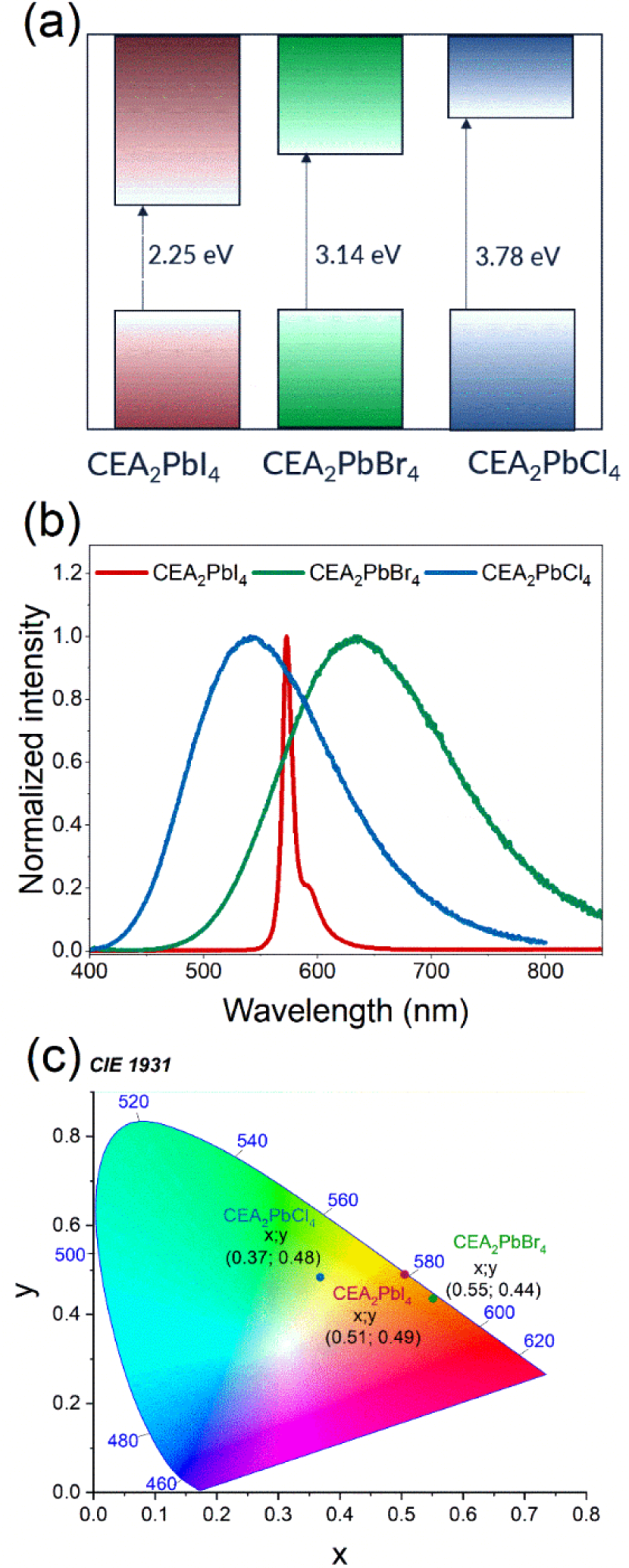
(a) Graphical presentation of the energy band gaps of the investigated compounds, (b) PL spectra of CEA_2_PbX_4_ at 80 K, and (c) CIE coordinates of samples’ PL.

The PL properties of hybrid perovskites are significantly dependent on the size of the energy band gap. Therefore, energy band gap (E_g_) engineering has become an effective method for modifying the optoelectronic properties of newly discovered lead halides.^13,49,50^ For the CEA_2_PbX_4_ series studied, where X = Cl, Br, and I, the excitonic absorption and absorption edge showed a red shift with increasing ionic size of the halide (Figure S16). The excitonic absorption was observed at 574, 414, and 337 nm for iodide, bromide, and chloride, respectively (Figure S16). In the case of the iodide, the excitonic absorption is red shifted even when compared to methylhydrazinium lead iodide, MHy_2_PbI_4_ (excitonic peak at 561 nm),^28^ and in the case of bromide and chloride, the excitonic peak is only slightly blue shifted compared to MHy_2_PbBr_4_ (excitonic peak at 423 nm)^20^ and MHy_2_PbCl_4_ (excitonic peak at 344 nm).^23^ Since MHy_2_PbX_4_ compounds are known to exhibit strongly reduced band gaps, diffuse spectra indicate reduced band gaps also for CEA_2_PbX_4_ analogues. Using the Kubelka–Munk equation with R as reflectance:^51^
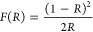
1and the Tauc modification, E_g_ of the obtained compounds was estimated as follows:^52^
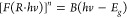
2where *h* and *v* denote the Planck constant and the photon’s frequency, while *B* is a constant; n is 1/2 or 2 for the direct and indirect band gaps, respectively. The smallest band gap (2.25 eV) is observed for CEA_2_PbI_4_ and it increases to 3.14 and 3.78 eV for the Br and Cl analogues, respectively (Figures 11a and S17). The values obtained are only slightly larger compared to MHy_2_PbX_4_ analogues (2.20, 3.02, and 3.75), which are known to exhibit strongly reduced band gaps,^20,23,28^ confirming reduced band gaps also for CEA_2_PbX_4_ compounds.

PL spectra of CEA_2_PbX_4_, recorded at 80 K, are presented in Figure 11b. The Cl and Br analogues show the presence of broadband emission with a maximum at 543 and 635 nm, respectively. Broadband emission with a large Stokes shift (Δ*S*) and large fwhm, usually attributed to STEs, has been reported for many 2D perovskites.^13,53−55^ The presence of the broadband PL with Δ*S* = 1.6 eV and fwhm = 0.61 eV for CEA_2_PbCl_4_ can be attributed to a very large out-of-plane octahedral distortion present in this compound. PL of CEA_2_PbI_4_ is composed of two narrow peaks (fwhm = 0.04 eV). The first band at 573 nm corresponds well to the FE absorption and can be, therefore, attributed to the recombination of FE. A similar FE-related peak was observed for EA_2_PbI_4_ (EA= ethanolammonium) at 546–547 nm (value at 10–15 K)^31,32^ or near 541 nm (value at 80 K)^33^ and for MHy_2_PbI_4_ at 561 nm (at 80 K).^28^ As can be noticed, the FE emission of CEA_2_PbI_4_ is red shifted compared to EA_2_PbI_4_ and MHy_2_PbI_4_, indicating that the band gap of these perovskites is even more strongly reduced than the band gaps of EA_2_PbI_4_ and MHy_2_PbI_4_. The second band is observed for CEA_2_PbI_4_ at 591 nm (Δ*S* = 0.18 eV). Similar bands were also reported for EA_2_PbI_4_ at 550–552 nm (value at 10–15 K)^31,32^ or near 551 nm (value at 80 K)^33^ and for MHy_2_PbI_4_ at 573 nm (at 80 K).^28^ This peak originates most likely either from the recombination of bound excitons (BE) or polaronic excitons.^28,33^ The presence of narrow PL is consistent with very small tilting of PbI_6_ octahedra in CEA_2_PbI_4_. The PL of CEA_2_PbBr_4_ is broad (fwhm = 0.52 eV) and strongly Stokes shifted (Δ*S* = 2.4 eV), i.e., it resembles that observed for the chloride in spite of small octahedral distortion. The origin of this unexpected behavior remains elusive. However, it is worth noting that BEA_2_PbBr_4_ and MHy_2_PbBr_4_ show the presence of both narrow FE-related PL (at 468 nm and 416 + 425 nm, respectively) as well as broadband STE emission (550–650 and 507 nm, respectively).^20,37^ Due to the presence of different emissions, the registered emission’s color changed from yellow-green for CEA_2_PbCl_4_ to yellow for CEA_2_PbBr_4_ and orange-yellow for CEA_2_PbI_4_ (Figure 11c). PL studies showed that under 360 nm excitation, the PLQY of CEA_2_PbBr_4_ is very high at 80 K (48%). Unfortunately, it was not possible to record PLQY for the remaining samples. Literature data show that PLQY values for 2D lead halide perovskites recorded at RT are of the order of fractions of a percent or a few percent. For instance, the recently reported PLQY of (N-MEDA)PbBr_4_ is 0.5% and it rises to 1.5% for the chloride analogue.^54^ In the case of (EDBE)PbBr_4_ perovskite, a large increase of PLQY to 9% was reported, but this compound crystallizes in the corrugated structure, representing [110] cuts of the perovskite structure.^56^ In order to fully identify the origin of the PL bands, femtosecond time-resolved measurements were performed at 80 K (Figures S18–S20). In the case of the studied perovskites, the emission decays rapidly with time ranging from 0.22 to 5.05 ns. All registered decay curves were composed of very fast and slower components, which decreased in the order Cl > Br > I. Comparable values of the emission decay times have been reported for many other 3D and 2D lead halide perovskites.^13,28,57,58^

**Figure 12:**
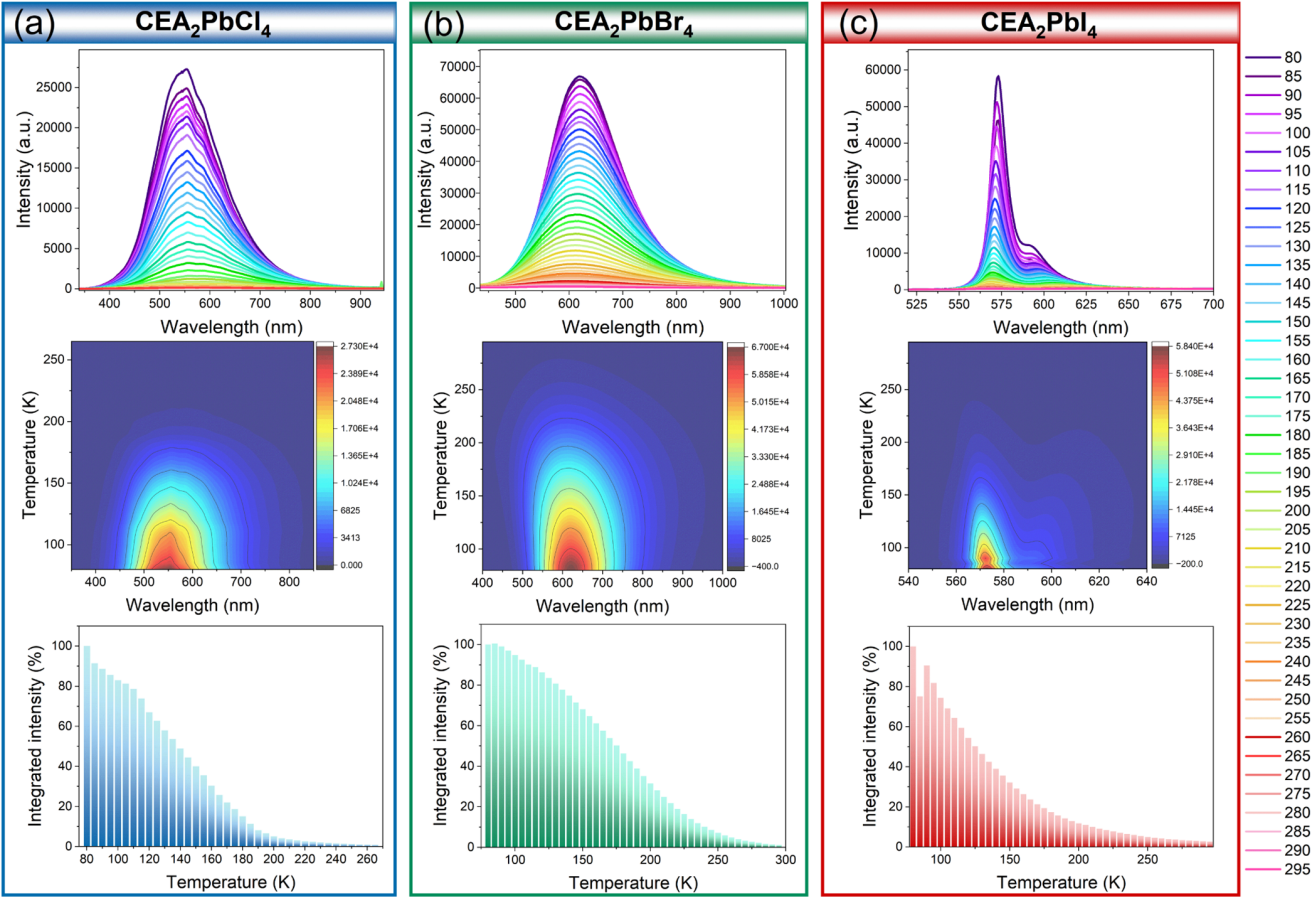
Temperature-dependent emission spectra, PL intensity contour maps, and changes of PL intensity in the function of the temperature of (a) CEA_2_PbCl_4_, (b) CEA_2_PbBr_4_, and (c) CEA_2_PbI_4_.

The temperature-dependent PL spectra of the investigated 2D compounds showed a monotonically decreasing PL intensity with sample heating (Figure 12). However, the position as well as the shape of the PL bands of CEA_2_PbCl_4_ and CEA_2_PbBr_4_ have not shown any significant changes in the whole temperature range studied. As a result, no change in the emission color could be observed by the naked eye. Different behavior is, however, observed for CEA_2_PbI_4_; namely, for this compound, a slight blue (red) shift from 573 to 566 nm (591 to 616 nm) is observed for the FE (BE) band with the increase in temperature (Figure 12c), leading to a change of the CIE coordinates from (0.51,0.49) at 80 K to (52,45) at 200 K. This change is small, and the color at 200 K is still orange-yellow. The different nature of PL is responsible for much faster quenching of CEA_2_PbI_4_ narrow FE and BE-related emission with a quenching temperature (T_0.5_) of 125 K, compared to the more stable broadband STE emission of the Cl and Br analogues, with T_0.5_ of 140 and 175 K, respectively.

## Conclusions

We have synthesized three layered lead halides comprising 2-chloroethylammonium cations: the previously reported iodide and the novel bromide and chloride. These compounds have been investigated using various experimental methods to monitor their crystal structures, mechanisms of PTs, and dielectric and optical properties.

Single-crystal X-ray diffraction revealed that at RT, the iodide and bromide are isostructural and crystallize in the ordered phases (space group *Pbnm*) with the CEA^+^ cations oriented parallel to the perovskite layers. Additionally, intermolecular N–H···Cl HBs occur between adjacent CEA^+^. On heating, CEA_2_PbBr_4_ and CEA_2_PbI_4_ undergo PT ∼315 K and ∼360 K, respectively, into the *Pbnm* phase associated with a disordering of CEA^+^ cations. Replacement of Br^–^ or I^–^ anions by Cl^–^ results in spectacular structural changes: (i) the orientation of CEA^+^ changes to perpendicular, resulting in a significant increase of the interlayer distance, (ii) the intermolecular HBs that stabilize the structure in the (001) planes in the bromide and iodide are replaced by Cl···Cl bonding, (iii) a pronounced increase in the in-plane octahedral tilting occurs. Due to these structural changes, CEA_2_PbCl_4_ exhibits not one but two PTs at 332 and 203 K. Furthermore, the RT phase remains partially disordered, and the degree of disorder of the HT phase is much greater than in the bromide, affecting both the inorganic and organic substructures.

The order–disorder nature of the HT PTs in the bromide and chloride has been confirmed by Raman and dielectric data through sudden broadening of Raman bands and a step-like increase of the dielectric permittivity at the PT temperatures. Although the crystal structure of the LT phase of CEA_2_PbCl_4_ could not be solved, the presence of the step-like anomaly near 200 K indicated locking of CEA^+^ motions below T_2_. This conclusion has been supported by the analysis of the Raman data, which also provided evidence that the ordering of CEA^+^ is associated with increased deformation of the inorganic substructure, symmetry decrease, and doubling of the number of unique CEA^+^ cations.

Optical studies revealed that all compounds exhibit efficient emission: broad band for bromide and chloride and narrow for iodide. Notably, the PLQY of CEA_2_PbBr_4_ (48%) is outstanding among lead bromides, representing [100] cuts of the perovskite structure. We have discussed the origin of these emissions and showed that the narrow emission of CEA_2_PbI_4_ is quenched much faster than the broadband emission observed for CEA_2_PbBr_4_ and CEA_2_PbCl_4_.

Overall, we have discovered that the presence or absence of intermolecular halogen bonding has a pronounced effect on the arrangement of halogenated organic cations, octahedral distortion, molecular dynamics, and phase stability. Thus, halide engineering is a powerful tool for tuning the optical and electrical properties of layered lead halide perovskites.

## Supporting Information Available

Additional experimental details, including selected hydrogen bond parameters, Raman and IR wavenumbers, photographs of the grown crystals, X-ray powder diffraction patterns, TG plots, DSC traces, reciprocal space reconstruction for the chloride, Raman, IR, dielectric and diffuse absorption spectra, Tauc plots, and decay curves (PDF)
