# Supplementary material for: Effect of Halide Tuning on the Structural, Dielectric, and Optical Properties of Two-Dimensional 2-Chloroethylammonium Lead Halides
Source: Inorg Chem. 2025 Feb 26;64(9):4501–13. doi: 10.1021/acs.inorgchem.4c05340 (PMC11898175; doi:10.1021/acs.inorgchem.4c05340)
Supplement: Supplementary file 1 — ic4c05340_si_001.pdf [file ic4c05340_si_001.pdf]

Supporting Information for

Effect of Halide Tuning on the Structural, Dielectric and Optical Properties of Two-Dimensional 2-chloroethylammonium Lead Halides

Mirosław Mączka,*[a] Jan Kudrawiec,[a] Katarzyna Fedoruk-Piskorska,[b] Dagmara Stefańska,[a] Anna Gągor,[a] Marek Drozd,[a] Szymon Smółka,[a] Adam Sieradzki[b]

[a]*Institute of Low Temperature and Structure Research, Polish Academy of Sciences, ul. Okólna 2, 50-422 Wrocław, Poland*

[b]*Department of Experimental Physics, Wrocław University of Science and Technology, Wybrzeże Wyspiańskiego 27, 50-370, Wrocław, Poland*

e-mail: m.maczka@intibs.pl

Table S1. Selected hydrogen-bond parameters in CEA_2PbX_4

D—H···*A*	*D*—H (Å)	H···*A* (Å)	*D*···*A* (Å)	*D*—H···*A* (°)
CEA_2PbBr_4 Phase II , 290K				
N1—H1A···Br1^{i}	0.89	2.82	3.548 (8)	140.6
N1—H1B···Br1^{ii}	0.89	2.56	3.406 (6)	159.2
N1—H1C···Br1	0.89	2.48	3.374 (6)	176.3
CEA_2PbCl_4 Phase II , 290K				
N1—H1A···Cl1^{iii}	0.89	2.37	3.228 (9)	162.3
N1—H1B···Cl1	0.89	2.40	3.268 (9)	166.0
N1—H1C···Cl2^{iii}	0.89	2.74	3.400 (10)	131.8
N1—H1C···Cl3	0.89	2.64	3.279 (11)	129.6

Symmetry code(s): (i) -x+1, -y+1, -z+1; (ii) x+1/2, -y+1/2; (iii) −*x*+1/2, *y*−1/2, *z*.

Table S2. RT Raman and IR wavenumbers (in cm^{-1}) of powdered CEA_2PbX_4 samples.

CEA_2PbCl_4		CEA_2PbBr_4		CEA_2PbI_4		assignment
RS	IR	RS	IR	RS	IR	
3173vw	3167vs	3194vw	3194m	3174vw	3167m	$\nu_{as}(NH_3)$
	3127vs	3152vw	3149m	3134vw	3125m	$\nu_{as}(NH_3)$
3062vw	3088s	3057vw	3073vs	3047vw	3052s	$\nu_s(NH_3)$
	3035s	3039vw	3016s	3026vw	3019s+3004s	$\nu_s(NH_3)$
3013m		3020m		3010m		$\nu_{as}(CH_2)$
2997m		3001m	2994s	2992m		$\nu_{as}(CH_2)$
2962s	2961ms	2966s	2963s	2957s	2952s	$\nu_s(CH_2)$
		2961s	2963vb	2951s		$\nu_s(CH_2)$
2893w		2922w	2913m	2915w	2911s	$\nu_s(CH_2)$
2862w	2817m	2869w	2809w	2866w	2848m	overtone/combination
		2842w		2836w		overtone/combination
1597vw	1592sh	1596w	1586m	1581sh	1567s	$\delta_{as}(NH_3)$
1575w	1581s	1581w	1577m	1574m		$\delta_{as}(NH_3)$
1501m	1496s	1474m	1473s	1472m	1466s	$\delta_s(NH_3)$
1456m	1455w	1442m	1440m	1441m	1437m	$\delta(CH_2)$
1441m	1439m	1429m	1427m	1427m	1424m	$\delta(CH_2)$
1391w	1390w	1389w	1388w	1386w	1383w	$\omega(CH_2)$
1322m	1321m	1322m	1322w	1319m	1316w	$\omega(CH_2)$
1282w	1281w	1278w	1285w	1276w	1280w	$\tau(CH_2)$
1240m	1238m	1237m	1235w	1233m	1229w	$\tau(CH_2)$
1105w	1103w	1101m	1099w	1096m	1095w	$\rho(CH_2)$
1085w	1082m	1075m	1072w	1070m	1068w	$\rho(CH_2)$
1043m	1042w	1036m	1034w	1029m	1026w	$\nu_{as}(CCN)$
950w	949w	944w	943w	936w	933w	$\rho(NH_3)$
891m	888m	880m	874m	873m	866m	$\nu_s(CCN)$

836m	834w	832m	828w	829m	826w	$\rho(CH_2)$
671s	671m	653s	653w	653s	649m	ν(CCl)
	665sh			650sh		ν(CCl)
459m	458w	452m	450v	448m	446vw	δ(CCN)
325w		327w		314w		δ(CCCl)
296w		293w		266w		$\tau(NH_3)$
180s						L+ T'+ Pb-Cl stretch
158s						L+ T'+ Pb-Cl stretch
129s		118s		89s		L+ T'+ Pb-X stretch
97m						Pb-Cl bend
87m		83vs		70sh		Pb-X bend
72sh		62s		60vs		Pb-X bend
62s		57s				Pb-X bend

key: vs, very strong; s, strong; m, medium; w, weak; sh, shoulder; ν, stretching; δ, bending (scissoring); ρ, rocking; ω, wagging; τ, twist; L, librational mode of organic cation; T', translational mode of organic cation

Table S3. RT Raman wavenumbers (in cm^{-1}) of CEA_2PbBr_4 for different polarization configurations.

z(xx)z - A_g	z(yy)z - A_g	y(zz)y - A_g	x(yz)x – B_{1g}	z(xy)z – B_{2g}	y(xz)y – B_{3g}	assignment
		3191w,b				$\nu_{as}(NH_3)$
3150vw	3150w	3150w,b	3145vw	3149vw	3149vw	$\nu_{as}(NH_3)$
	3060vw,b		3061w,b	3054w	3057w,b	$\nu_s(NH_3)$
			3036w,b	3035w,b	3035w,b	$\nu_s(NH_3)$
3020w	3020w	3020w	3019w	3020w	3019w	$\nu_{as}(CH_2)$
3001w	3001w	3001w	3001w	3000m	3001m	$\nu_{as}(CH_2)$
2966s	2966m	2966s	2966s	2966s	2966s	$\nu_s(CH_2)$
2960sh	2960sh	2960sh	2959sh	2959sh	2959sh	$\nu_s(CH_2)$
2923vw	2923vw	2923vw	2920w	2922w	2921w	$\nu_s(CH_2)$
1595vw	1598w+1593w	1595vw	1593vw	1595w	1595w	$\delta_{as}(NH_3)$
1581m	1581m	1581w	1581m	1581m	1581m	$\delta_{as}(NH_3)$
1475w	1475w	1475w	1473m	1473vw	1474w	$\delta_s(NH_3)$
1443m	1443m	1443w	1442m	1443w	1442m	$\delta(CH_2)$
1429m	1429m	1429w	1429m	1429m	1429m	$\delta(CH_2)$
1389w	1389w	1389w	1389w	1389w	1390w	$\omega(CH_2)$
1322m	1322m	1322w	1322m	1322m	1322m	$\omega(CH_2)$
1278w	1278w	1278w	1283sh+1277w	1281w+1277w	1281w	$\tau(CH_2)$
1237w	1237w	1237w	1236w	1236w	1236w	$\tau(CH_2)$
1102w	1102m	1102w	1102m	1101m	1102m	$\rho(CH_2)$
1075w	1075m	1075w	1074m	1075m	1074m	$\rho(CH_2)$
1036w	1036w	1036w	1035w	1035w	1036w	$\nu_{as}(CCN)$
944m	944w	942w	944w	943w	943w	$\rho(NH_3)$
878m	878m	878m	879m	878m	879m	$\nu_s(CCN)$

832s	832m	832m	931w	933w	932w	ρ(CH_2)
653s	653m	653s	653s	653w	653s	ν(CCl)
452w	452w	452w	452w	453w	452w	δ(CCN)
326m,b	326w,b	326w,b	327w,b	327w+320w	325w,b	δ(CCCl)
		290w,b	289w,b	273w	281w,b	τ(NH_3)+δ(CCCl)
118vs	118vs	118s	118s	118s	118s	L+ T'+ Pb-Br stretch
84vs	84vs	84vs	81vs	86vs	84vs	Pb-Br bend
		62vs	62vs		62vs	Pb-Br bend
56vs	56vs	56vs	57vs	55vs	57vs	Pb-Br bend

key: vs, very strong; s, strong; m, medium; w, weak; sh, shoulder; ν, stretching; b, broad; δ, bending (scissoring); ρ, rocking; ω, wagging; τ, twist; L, librational mode of organic cation; T', translational mode of organic cation

Table S4. Raman wavenumbers (in cm^{-1}) of CEA_2PbBr_4 at 80, 300 and 360 K.

80K	300 K	360 K	assignment
3187vw			$\nu_{as}(NH_3)$
3152w	3151w,b	3154w,b	$\nu_{as}(NH_3)$
3056m	3056w,b	3063w,b	$\nu_s(NH_3)$
3043m	3034w,b	3037w,b	$\nu_s(NH_3)$
3021m	3020w	3018w	$\nu_{as}(CH_2)$
2998m	2999w	3001w	$\nu_{as}(CH_2)$
2968s	2967s	2964s	$\nu_s(CH_2)$
2958s	2958sh	2957sh	$\nu_s(CH_2)$
2929m	2922w	2917w	$\nu_s(CH_2)$
1600w	1595vw		$\delta_{as}(NH_3)$
1581s	1580m	1580m	$\delta_{as}(NH_3)$
1476w	1474w	1473w	$\delta_s(NH_3)$
1443m	1442m	1442m	$\delta(CH_2)$
1427s	1428m	1428m	$\delta(CH_2)$
1398vw+1388w	1388w	1388w	$\omega(CH_2)$
1321m	1320w	1320w	$\omega(CH_2)$
1288vw+1276w	1277w	1279w	$\tau(CH_2)$
1237w	1236w	1235w	$\tau(CH_2)$
1101m	1100m	1101m	$\rho(CH_2)$
1076s	1074m	1072m	$\rho(CH_2)$
1036w	1035w	1035w	$\nu_{as}(CCN)$
947w	942w	937w,b	$\rho(NH_3)$
877m+880m	877m	877m	$\nu_s(CCN)$
835m	832m	829m	$\rho(CH_2)$
650m+646sh	652m	654m	$\nu(CCl)$

620vw			combination
452vw	452vw	452vw	δ(CCN)
335m	328sh		δ(CCCl)
307sh+296m	290m,b	286m,b	τ(NH_3)+δ(CCCl)
275sh			τ(NH_3)
181w			L+ T’
154w			L+ T’
121vs	116vs	120sh	L+ T’+ Pb-Br stretch

key: vs, very strong; s, strong; m, medium; w, weak; sh, shoulder; ν, stretching; b, broad; δ, bending (scissoring); ρ, rocking; ω, wagging; τ, twist; L, librational mode of organic cation; T’, translational mode of organic cation

Table S5. Raman wavenumbers (in cm^{-1}) of CEA_2PbI_4 at 80, 300 and 390 K.

80K	300 K	360 K	assignment
1582w+1576s	1575m	1574w	$\delta_{as}(NH_3)$
1474w	1471w	1466w	$\delta_s(NH_3)$
1424s	1427m	1429w	$\delta(CH_2)$
1389w+1385w	1385w	1382vw	$\omega(CH_2)$
1319m+1316w	1319m+1316sh	1318w	$\omega(CH_2)$
1278vw+1273w	1275w	1277vw	$\tau(CH_2)$
1234s	1233m	1232w	$\tau(CH_2)$
1095m	1095m	1096m	$\rho(CH_2)$
1072s	1070m	1065m	$\rho(CH_2)$
1028m	1028m	1028m	$\nu_{as}(CCN)$
938w	932vw		$\rho(NH_3)$
873s+870w	872m	872m	$\nu_s(CCN)$
831s	828m	825m	$\rho(CH_2)$
650m+646w	652m+648w	654m	$\nu(CCl)$
446m	448m	449m	$\delta(CCN)$

key: vs, very strong; s, strong; m, medium; w, weak; sh, shoulder; ν, stretching; b, broad; δ, bending (scissoring); ρ, rocking; ω, wagging; τ, twist; L, librational mode of organic cation; T', translational mode of organic cation

Table S6. Raman wavenumbers (in cm^{-1}) of CEA_2PbCl_4 at 80, 300 and 360 K.

80K	300 K	360K	assignment
1602vw			$\delta_{as}(NH_3)$
1582vw	1583vw,b	1583vw,b	$\delta_{as}(NH_3)$
1514vw+1502w	1499vw,b	1490vw,b	$\delta_s(NH_3)$
1463w+1456w	1455w	1454w	$\delta(CH_2)$
1444w+1334w	1440w	1440w	$\delta(CH_2)$
1392vw	1392vw	1391vw	$\omega(CH_2)$
1322m	1322m	1322m	$\omega(CH_2)$
1284w	1281w	1280w	$\tau(CH_2)$
1241w	1241w	1237w	$\tau(CH_2)$
1108m	1104w	1102w	$\rho(CH_2)$
1092vw+1085w	1084vw		$\rho(CH_2)$
1044m	1043m	1041m	$\nu_{as}(CCN)$
951m	950w	944vw,b	$\rho(NH_3)$
896m+893m	889m	885m	$\nu_s(CCN)$
835m	834m	833m	$\rho(CH_2)$
668s	671s	669s	$\nu(CCl)$
464w+457w	459w	457w	$\delta(CCN)$
339w+324w	327w		$\delta(CCCl)$
299w	292w	288sh	$\tau(NH_3)$
212w+197s	184s	183s,b	L+ T'+ Pb-Cl stretch
179s			L+ T'+ Pb-Cl stretch
164s+149s	155sh		L+ T'+ Pb-Cl stretch
129vs+115vs	125vs	123vs	L+ T'+ Pb-I stretch

key: vs, very strong; s, strong; m, medium; w, weak; sh, shoulder; b, broad; ν, stretching; δ, bending (scissoring); ρ, rocking; ω, wagging; τ, twist; L, librational mode of organic cation; T', translational mode of organic cation

Table S7. IR wavenumbers (in cm^{-1}) of CEA_2PbCl_4 at 7 and 300 K.

7K	300 K	assignment
3184s+3176sh+3170s	3177s	$\nu_{as}(NH_3)$
3156s+3146s+3136s	3137s	$\nu_{as}(NH_3)$
3096m+3083m+3073m+3068m	3087s	$\nu_s(NH_3)$
3036s+3010m+2998s	3031s	$\nu_s(NH_3)$
1611w+1601w	1594sh	$\delta_{as}(NH_3)$
1583s+1580s	1580m	$\delta_{as}(NH_3)$
1515s+1508w+1502s	1498s	$\delta_s(NH_3)$
1432m	1438m	$\delta(CH_2)$
1321m	1321m	$\omega(CH_2)$
1287w+1285w	1281w	$\tau(CH_2)$
1242w	1239w	$\tau(CH_2)$
1108sh+1105w	1103w	$\rho(CH_2)$
1093m+1083w	1083w	$\rho(CH_2)$
1044m	1042m	$\nu_{as}(CCN)$
955w	948w	$\rho(NH_3)$
896m+893m	890m	$\nu_s(CCN)$
834w	834w	$\rho(CH_2)$
669sh+666m+662w	671m+666m	$\nu(CCl)$
466w+459w	459w	$\delta(CCN)$

key: vs, very strong; s, strong; m, medium; w, weak; sh, shoulder; ν, stretching; δ, bending (scissoring); ρ, rocking; ω, wagging; τ, twist; L, librational mode of organic cation; T’, translational mode of organic cation

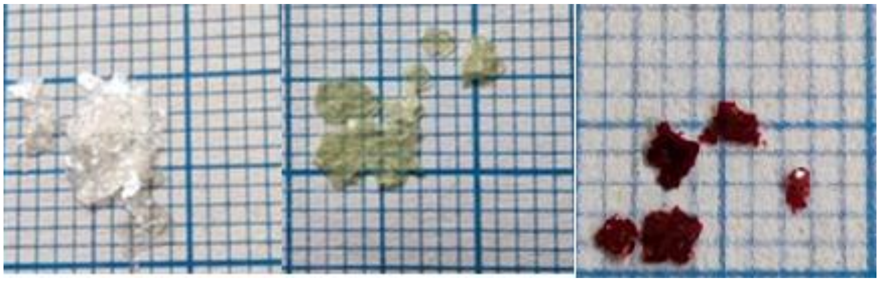

Figure S1. Photos of the CEA_2PbCl_4, CEA_2PbBr_4 and CEA_2PbI_4 crystals (from left to right).

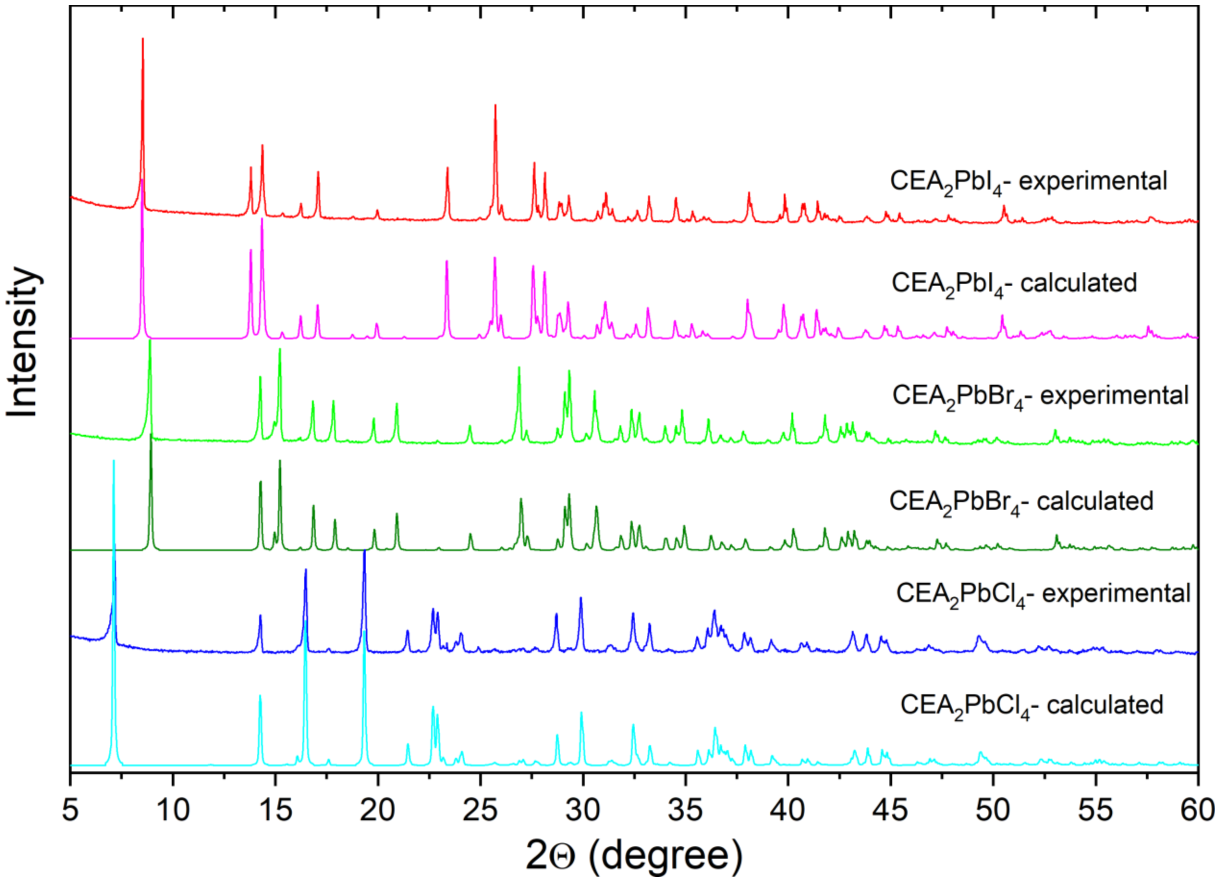

Figure S2. Experimental and simulated powder diffraction patterns of CEA_2PbX_4 samples (X=Cl, Br, I).

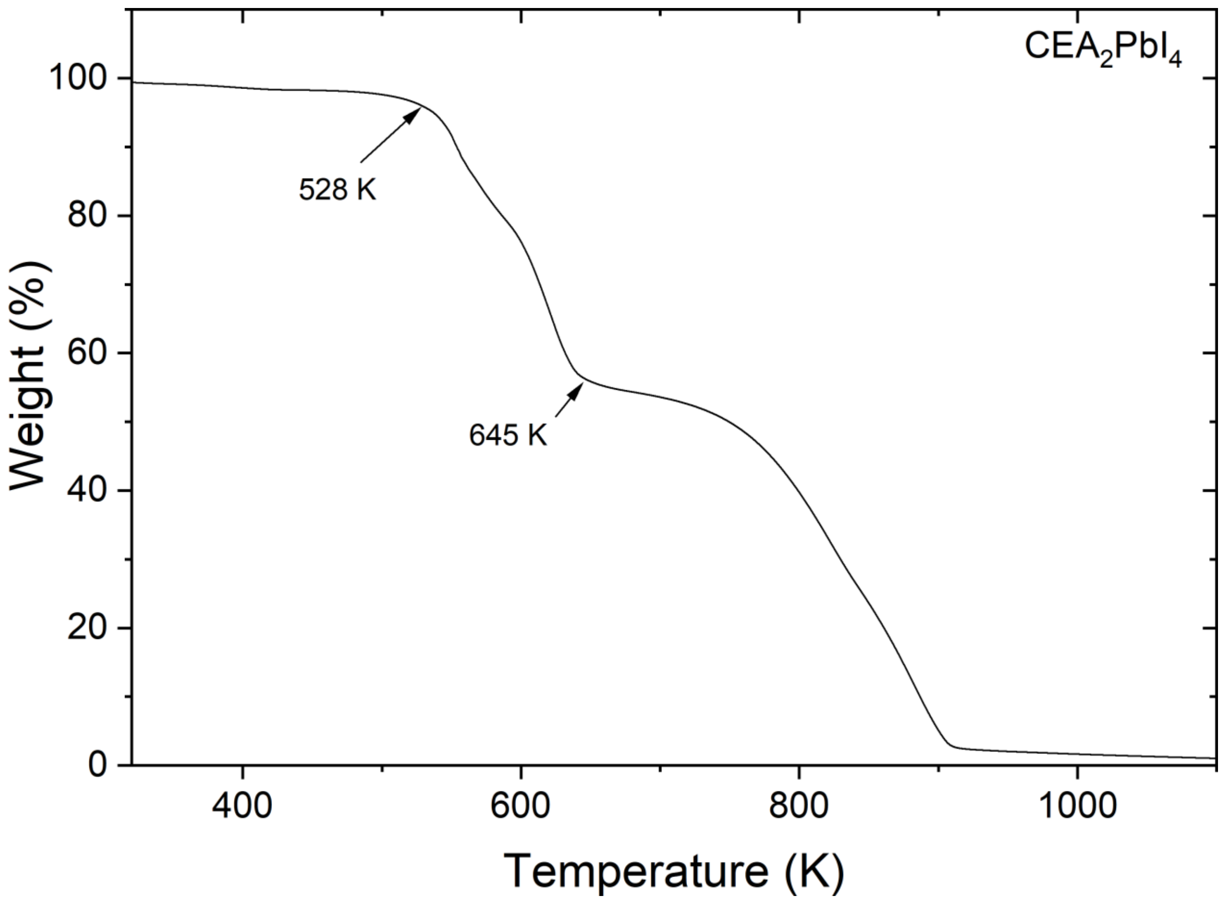

Figure S3. TG plot of CEA_2PbI_4.

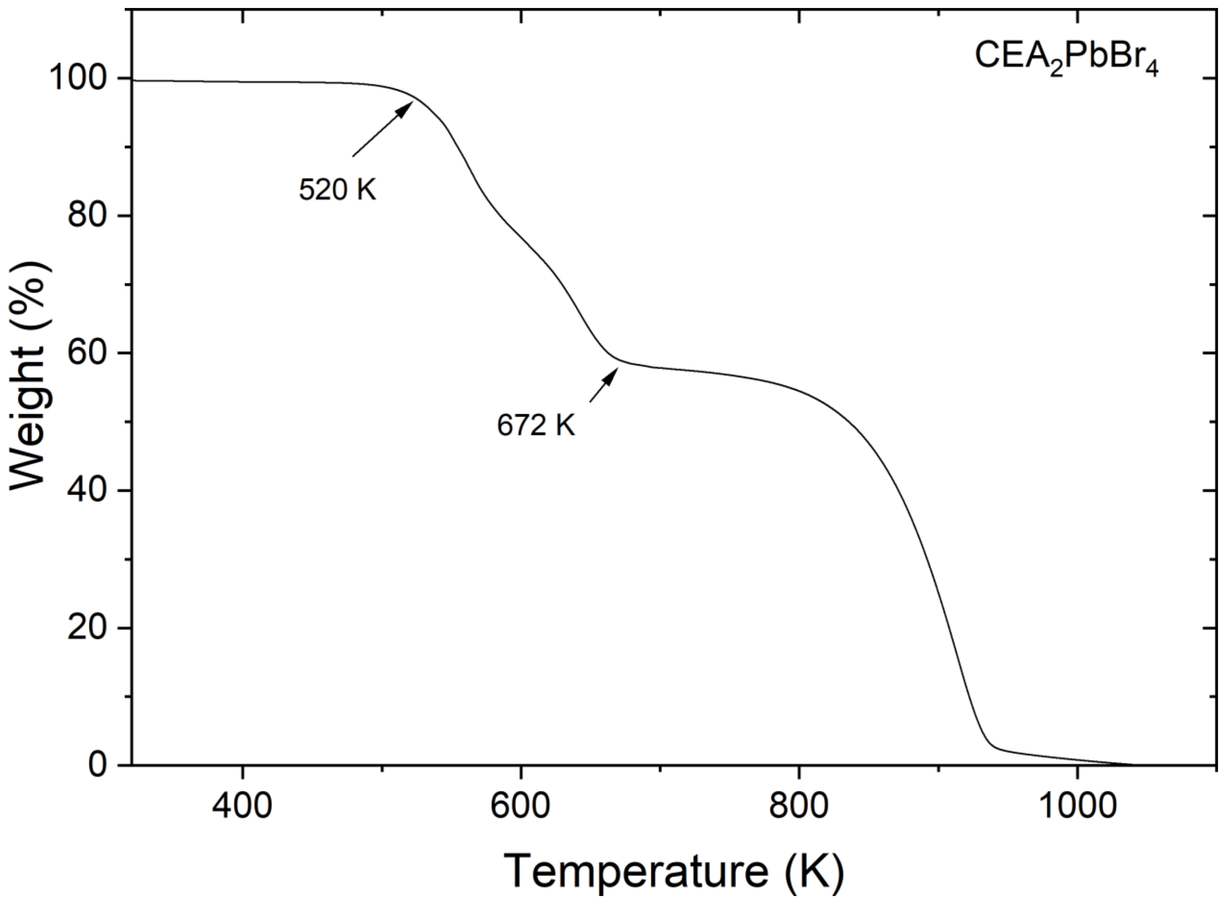

Figure S4. TG plot of CEA_2PbBr_4.

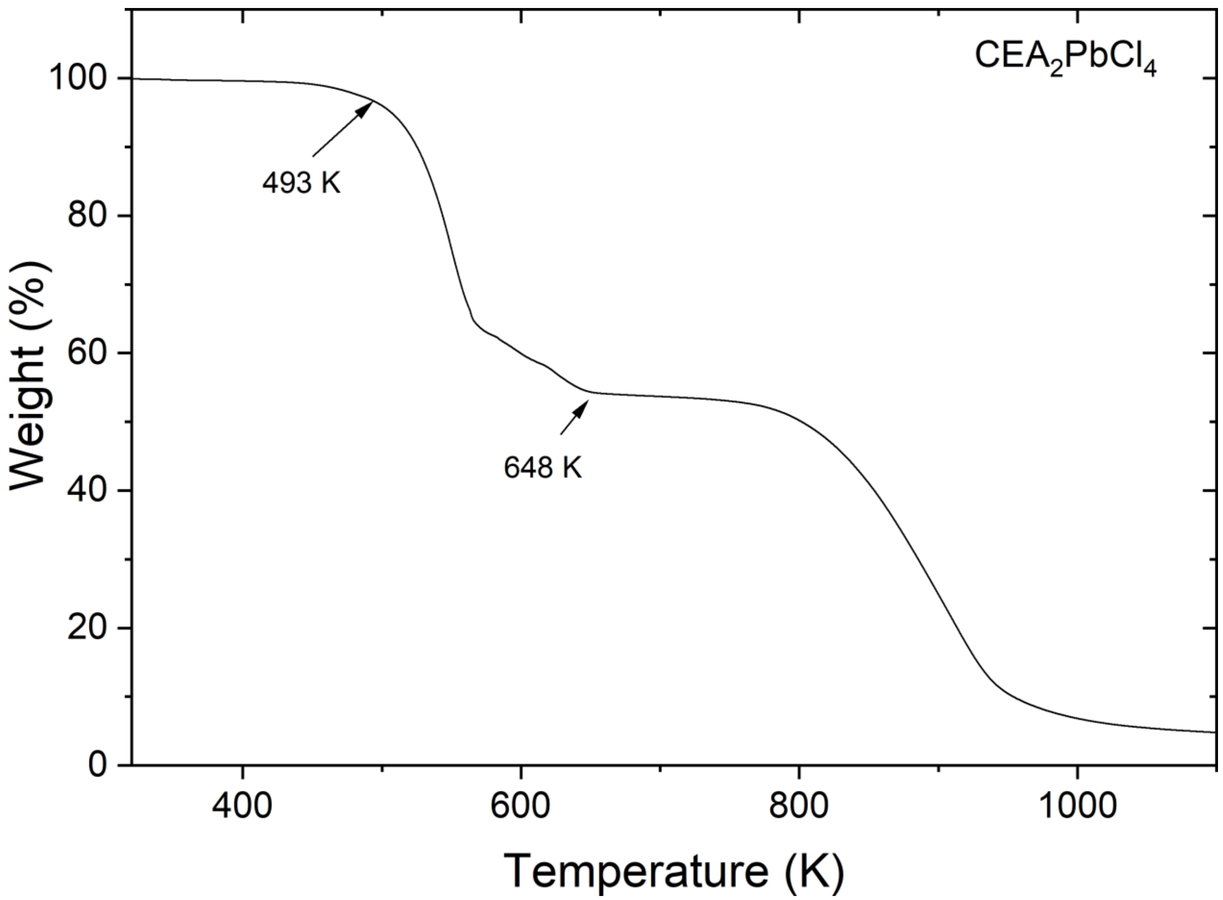

Figure S5. TG plot of CEA_2PbCl_4.

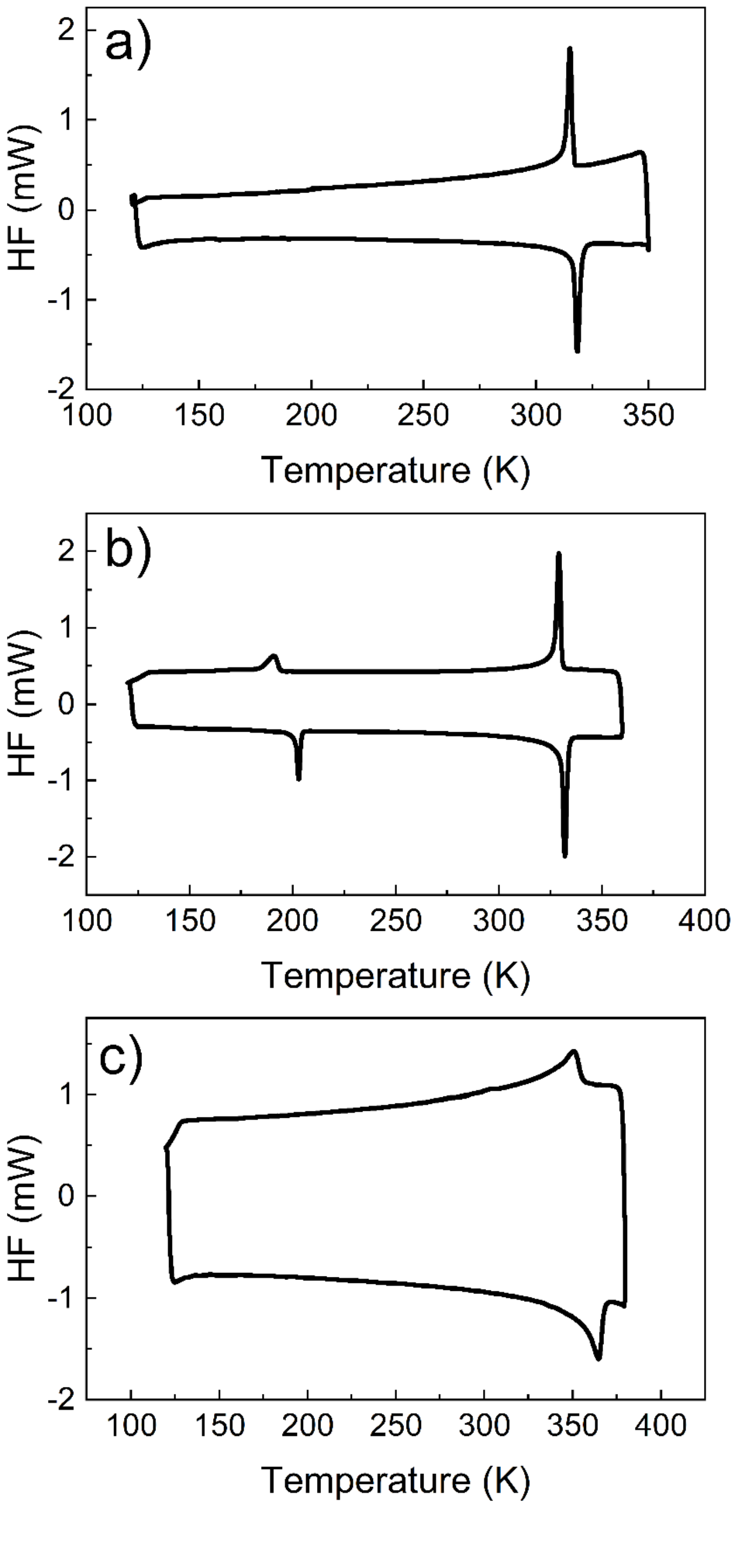

Figure S6. DSC traces for (a) CEA_2PbBr_4 , (b) CEA_2PbCl_4 and CEA_2PbI_4.

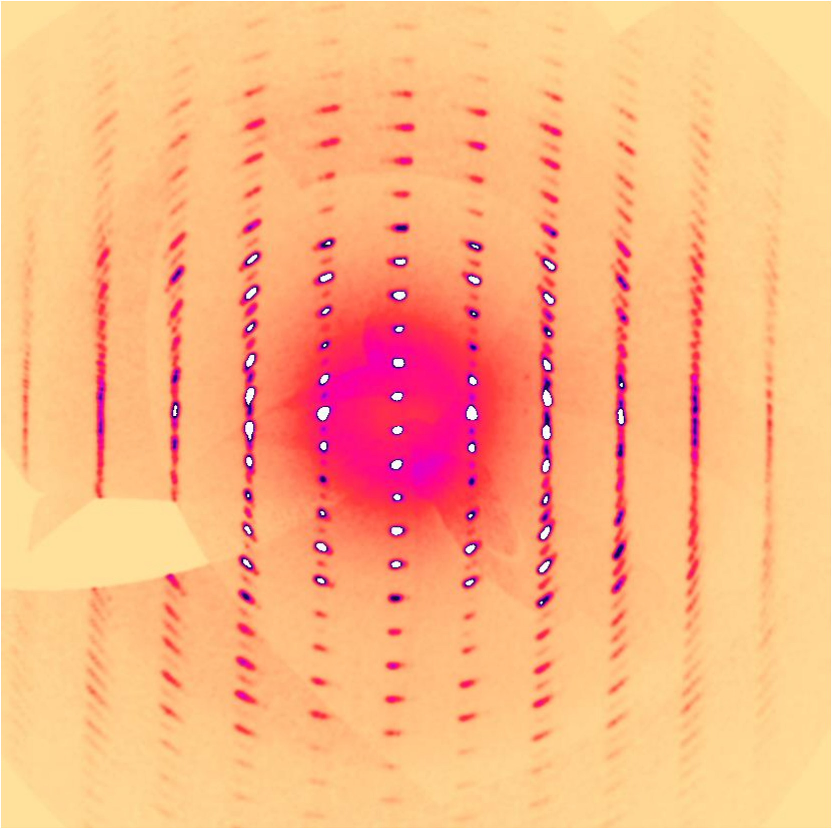

Figure S7. The reconstruction of the reciprocal 1kl plane in CEA_2PbCl_4, Phase III, T=120K. Splitting and blurring of diffraction peaks indicate significant symmetry reduction and the appearance of internal strain related to the change in the packing of the crystal structure.

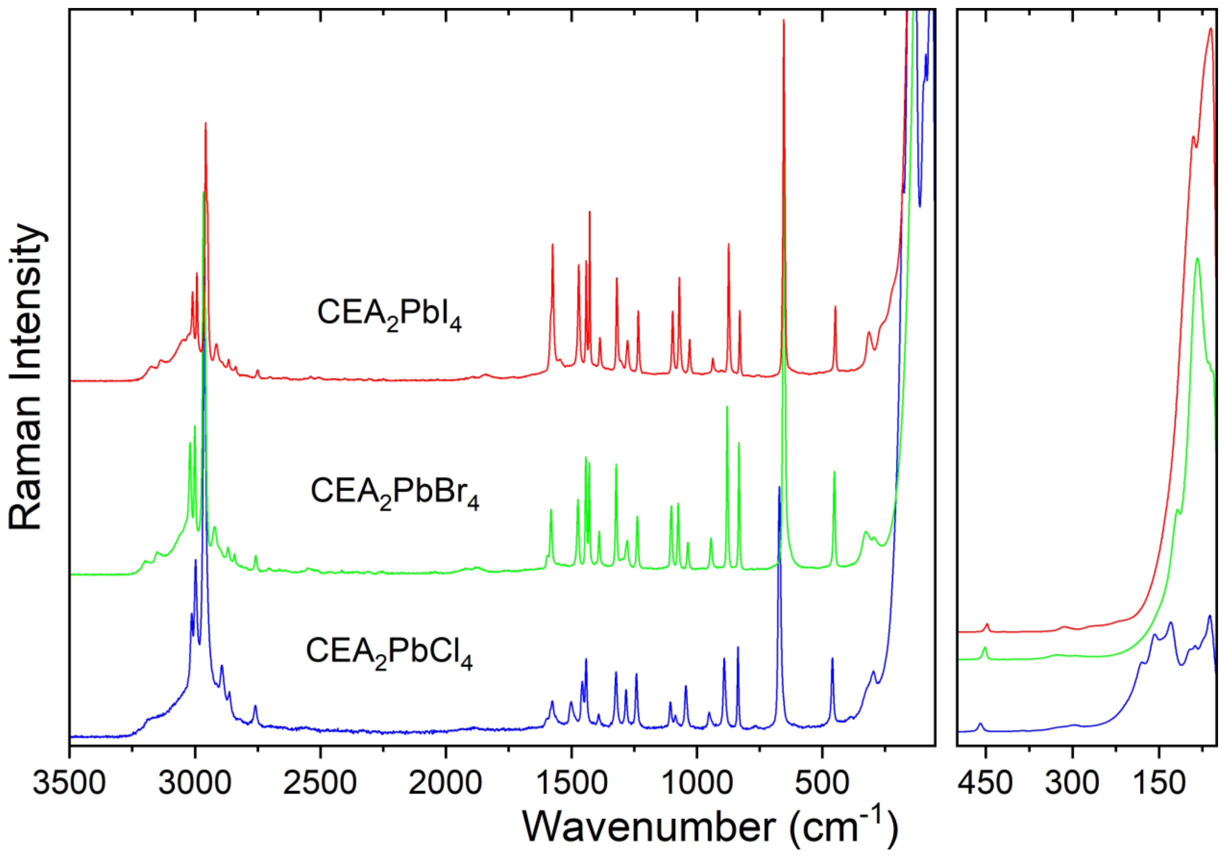

Figure S8. RT Raman spectra of CEA_2PbX_4 powdered samples in the 3500-50 cm^{-1} range.

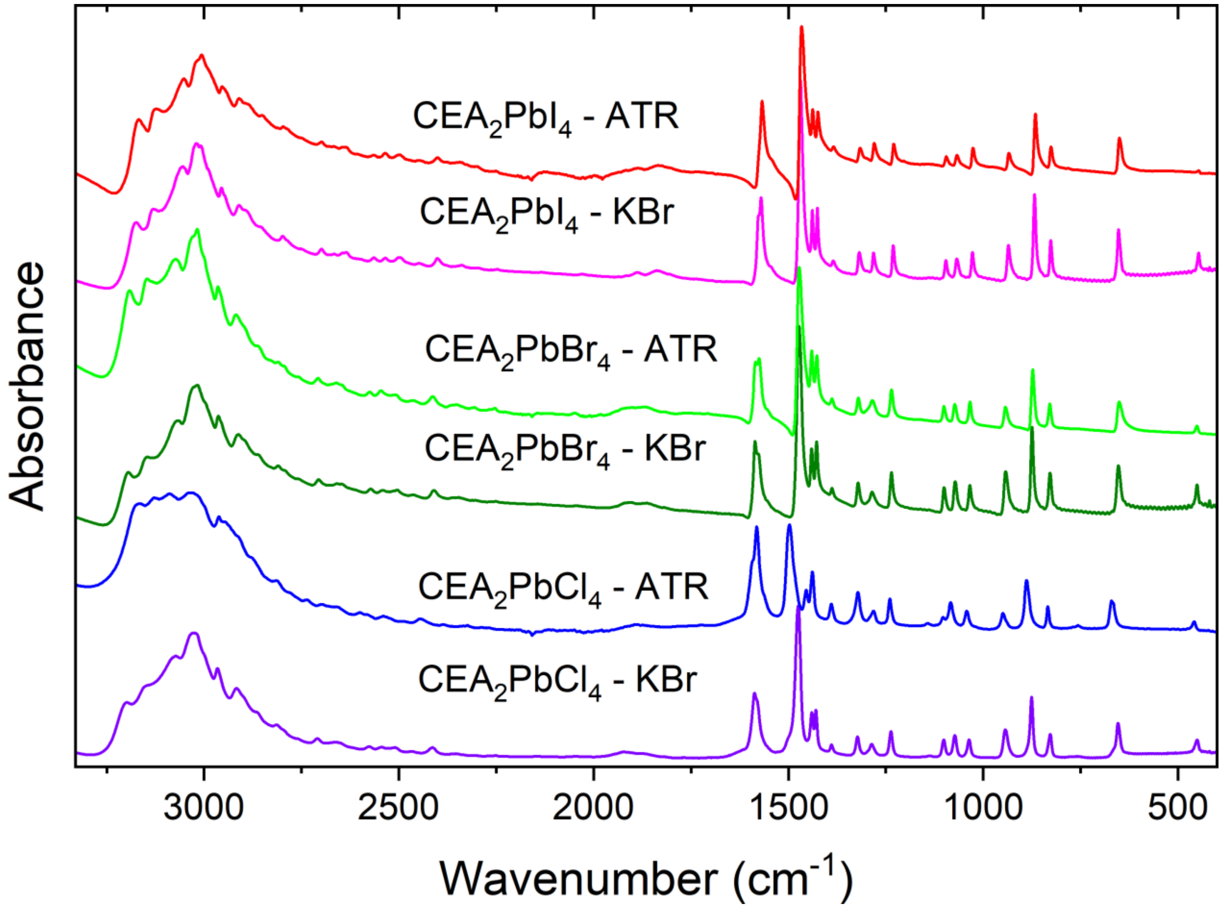

Figure S9. RT IR spectra of CEA_2PbX_4 powdered samples in the 3500-400 cm^{-1} range.

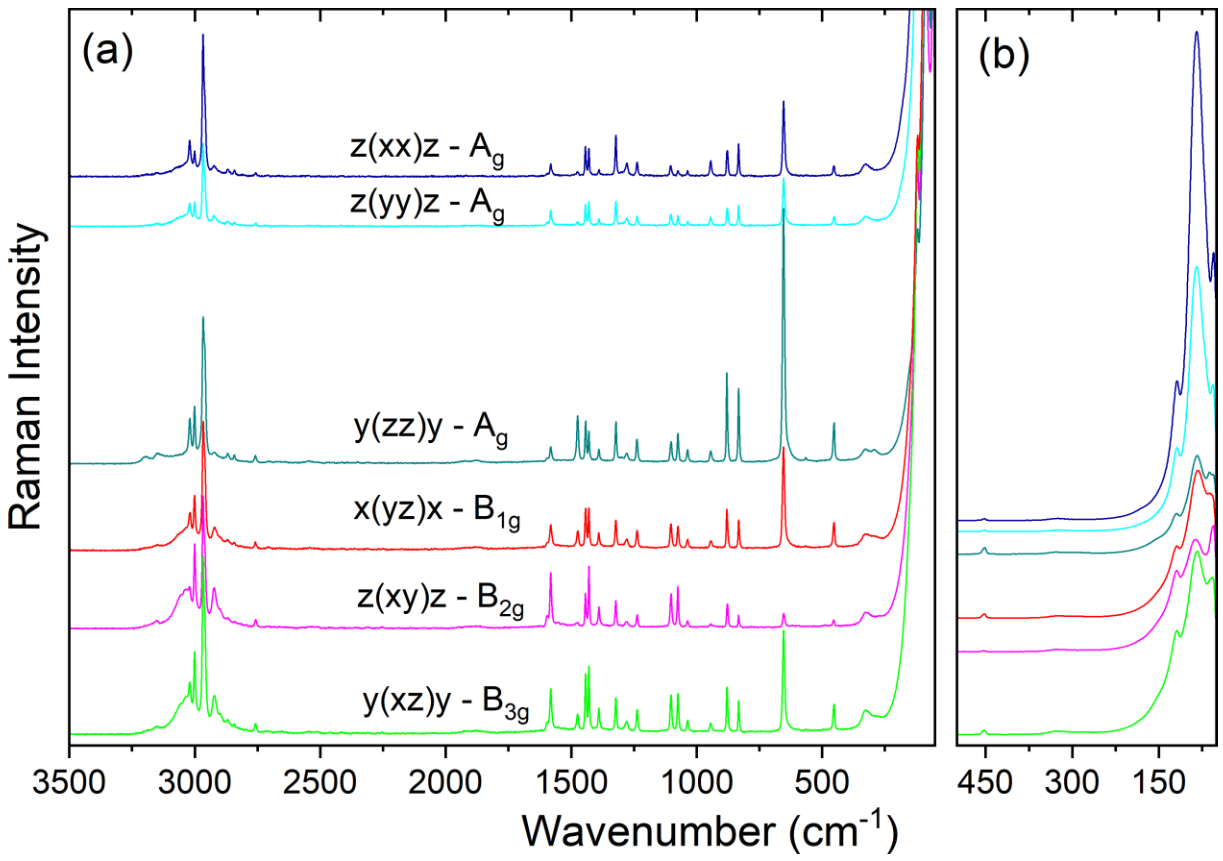

Figure S10. Polarized Raman spectra of CEA_2PbBr_4 powdered samples in the (a) 3500-500 cm^{-1} and (b) 500-50 cm^{-1} range.

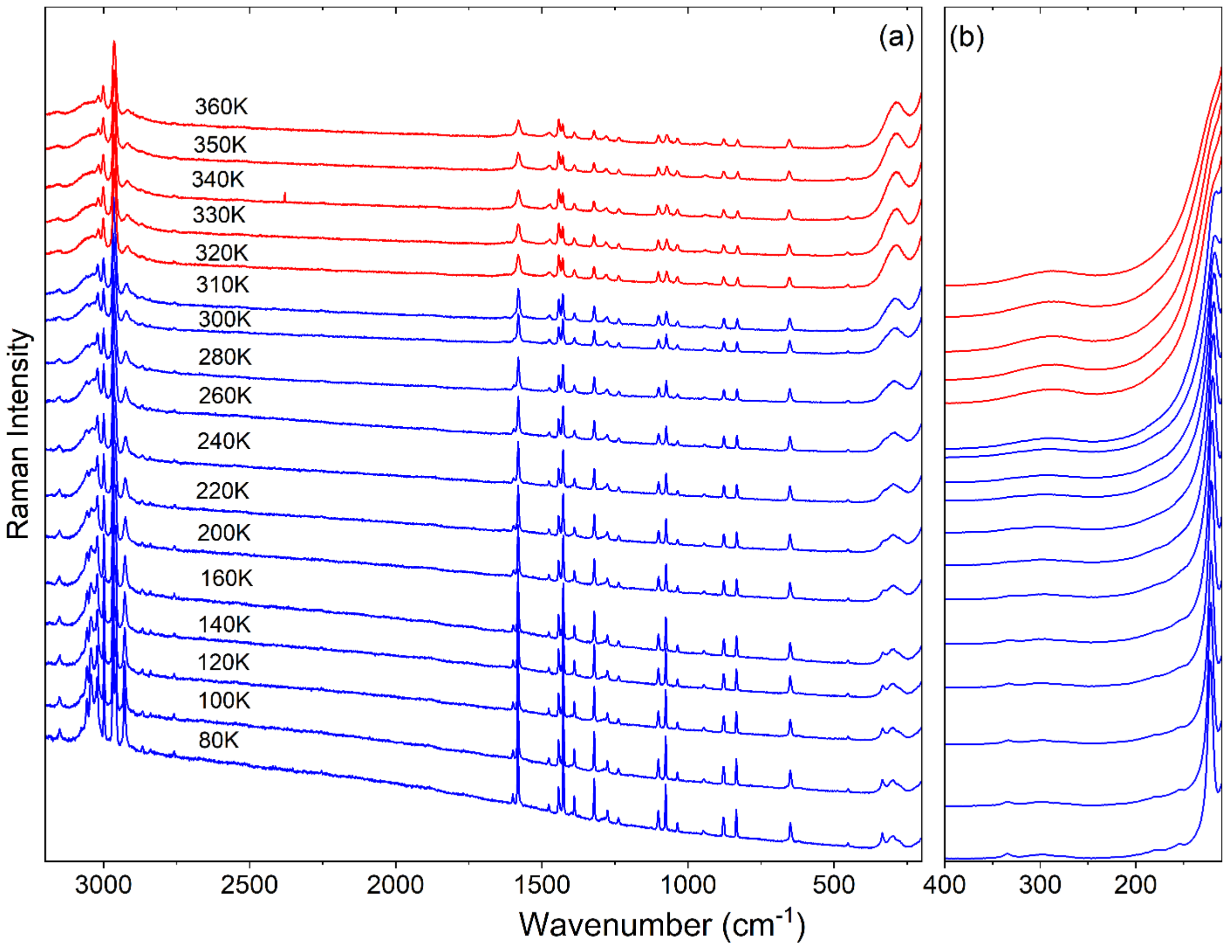

Figure S11. Raman spectra of CEA_2PbBr_4 in the (a) 3200-200 cm^{-1} and (b) 400-110 cm^{-1} range recorded on heating. Red and blue colours correspond to the HT and LT phase, respectively.

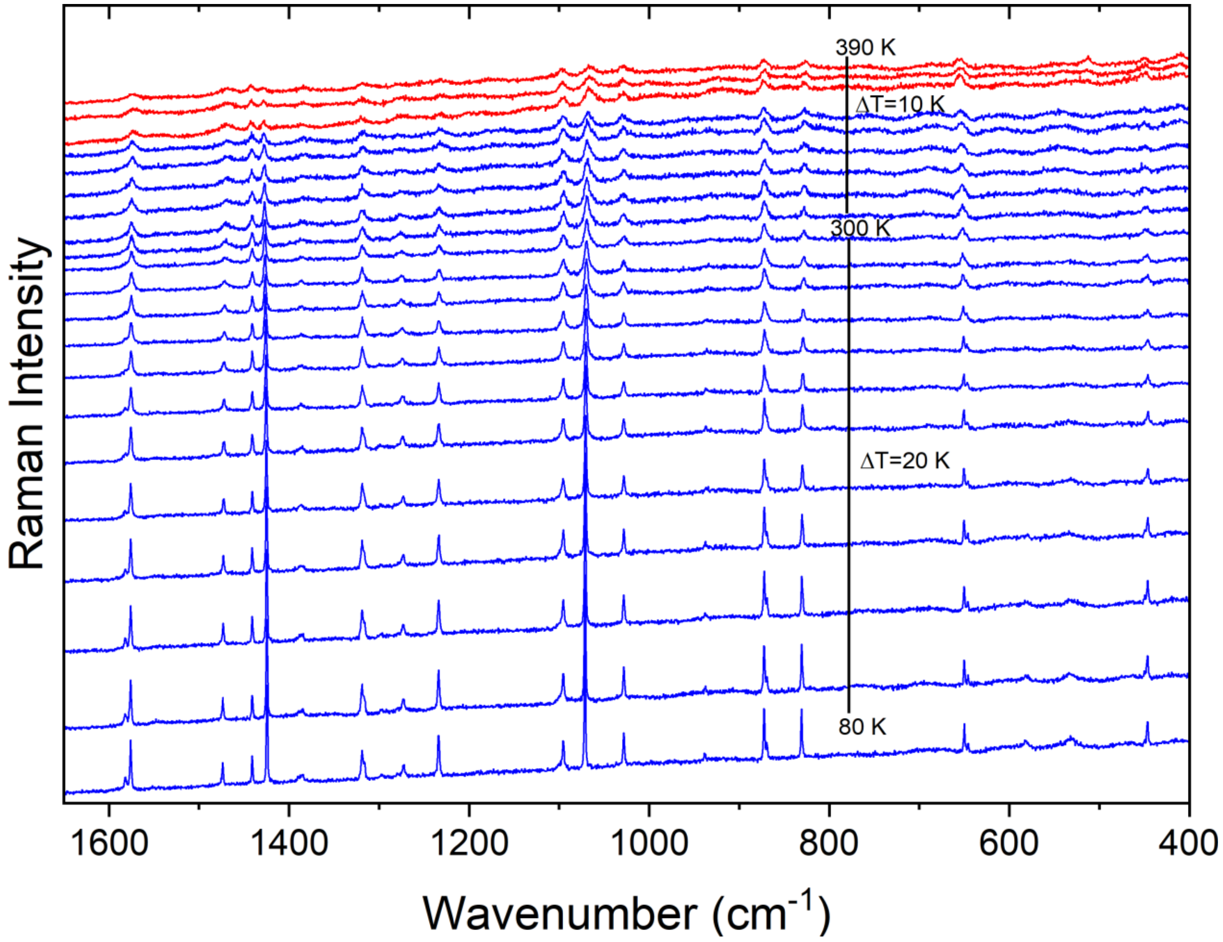

Figure S12. Raman spectra of CEA_2PbI_4 in the 1650-400 cm^{-1} range recorded on heating. Red and blue colours correspond to the HT and LT phase, respectively.

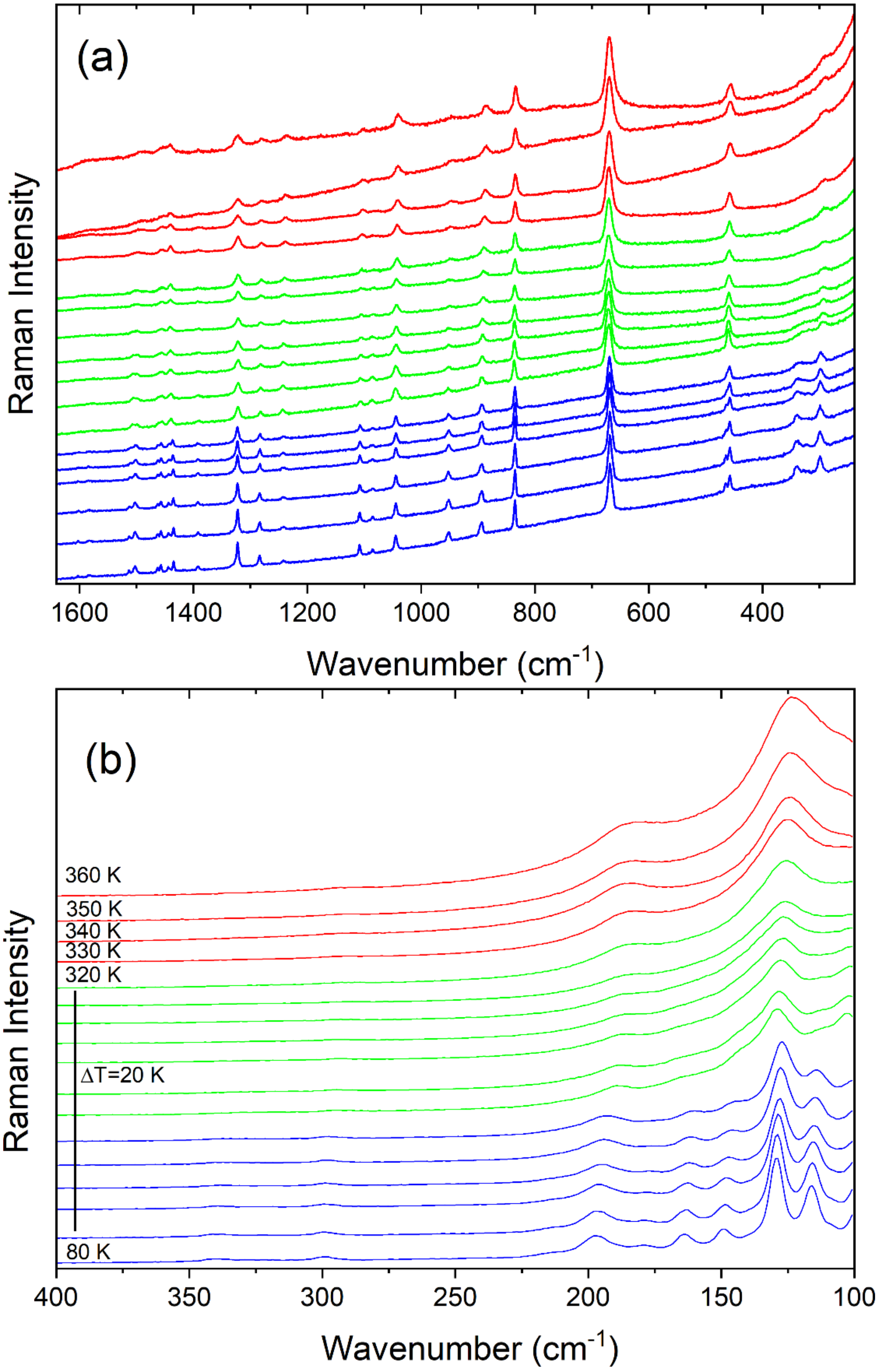

Figure S13. Raman spectra of CEA_2PbCl_4 in the 1650-100 cm^{-1} range recorded on heating. Red, green and blue colours correspond to the HT, intermediate and LT phase, respectively. Colour code is the same as in Fig. 7.

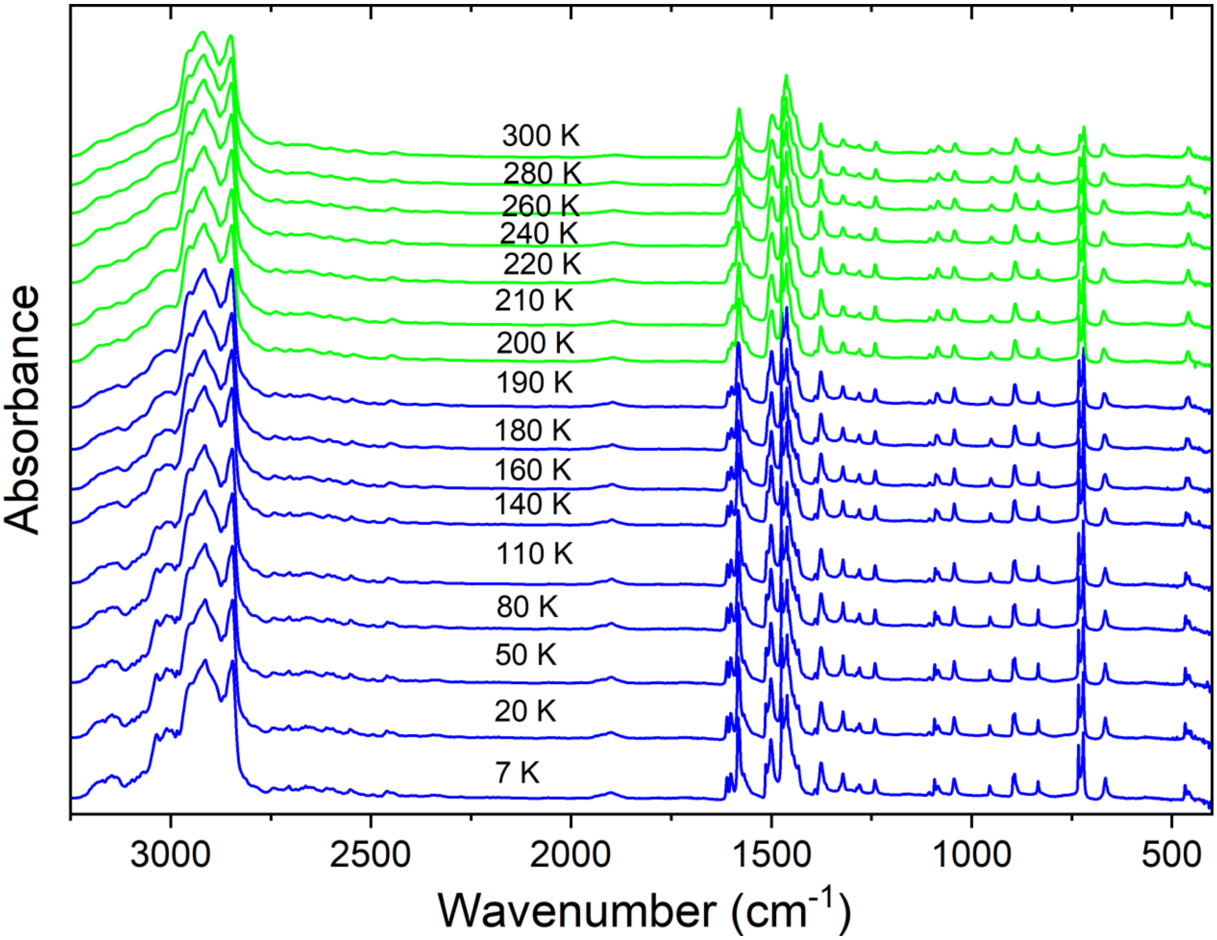

Figure S14. IR spectra of CEA_2PbCl_4 in the 3250-400 cm^{-1} range recorded on heating. Green and blue colours correspond to the intermediate and LT phase, respectively. Colour code is the same as in Fig. 8.

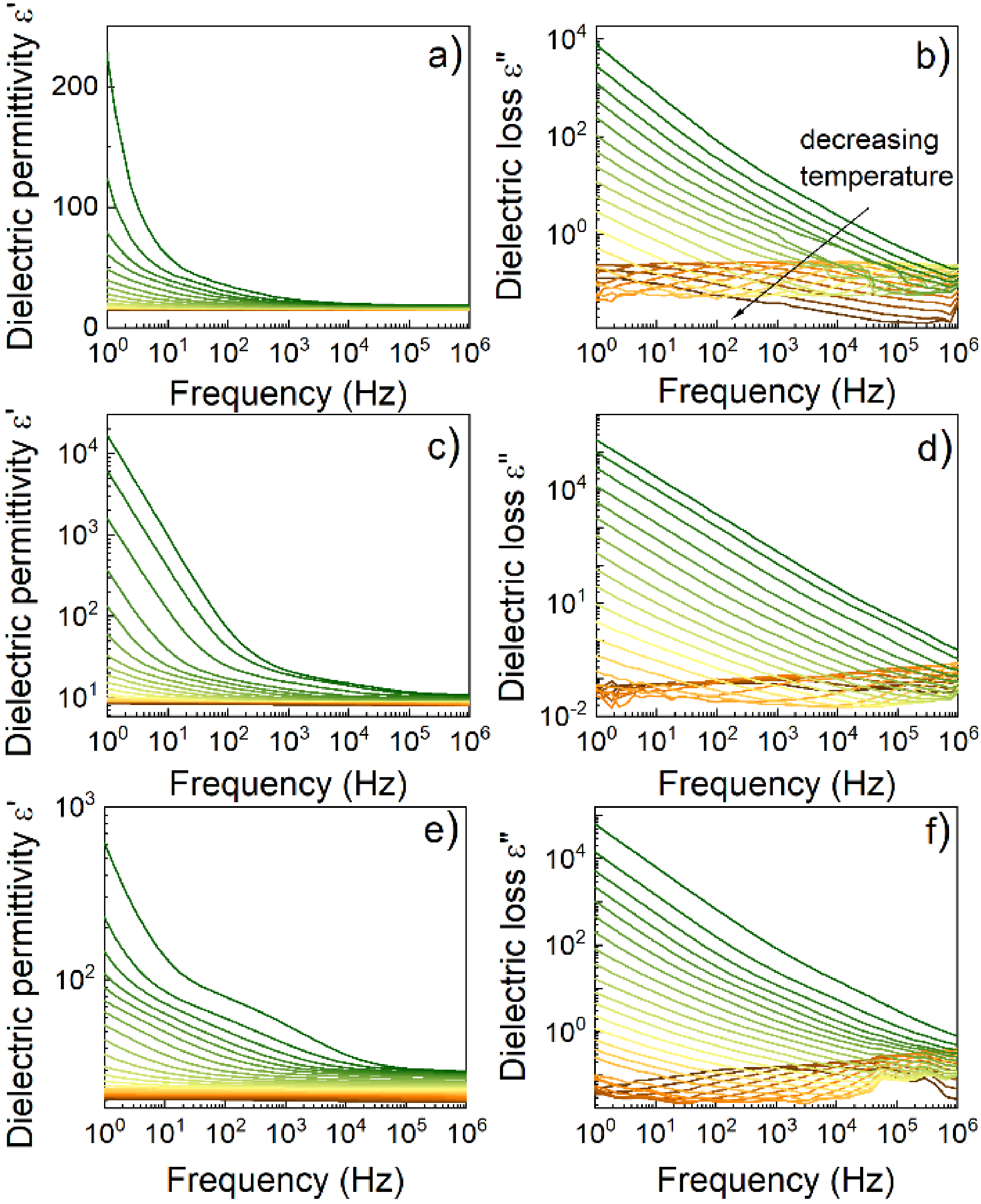

Figure S15. Frequency dependence of the complex dielectric permittivity for CEA_2PbBr_4 (a,b), CEA_2PbCl_4 (c,d) and CEA_2PbI_4 (e,f).

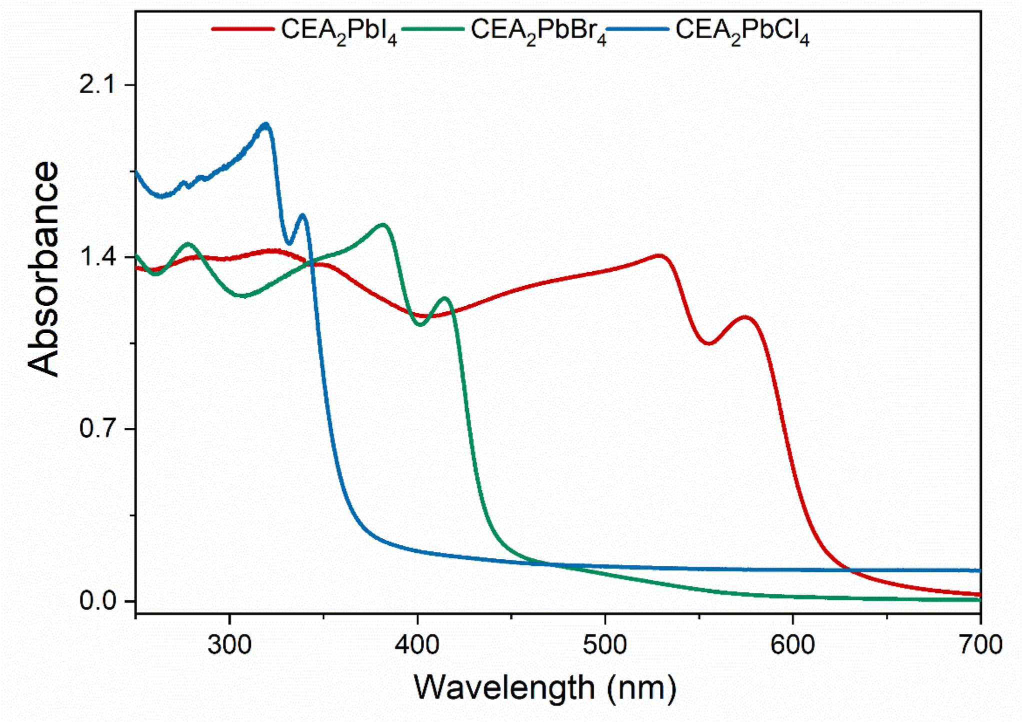

Figure S16. Diffuse absorption spectra of investigated perovskites.

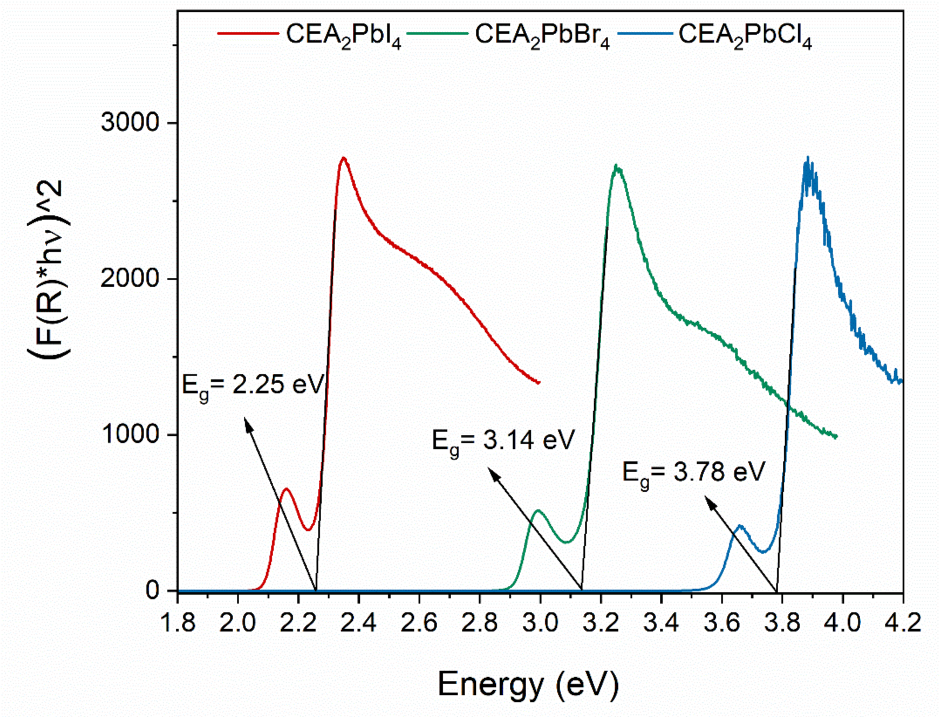

Figure S17. Energy band gap of the CEA_2PbX_4 samples determined using Tauc plot modification.

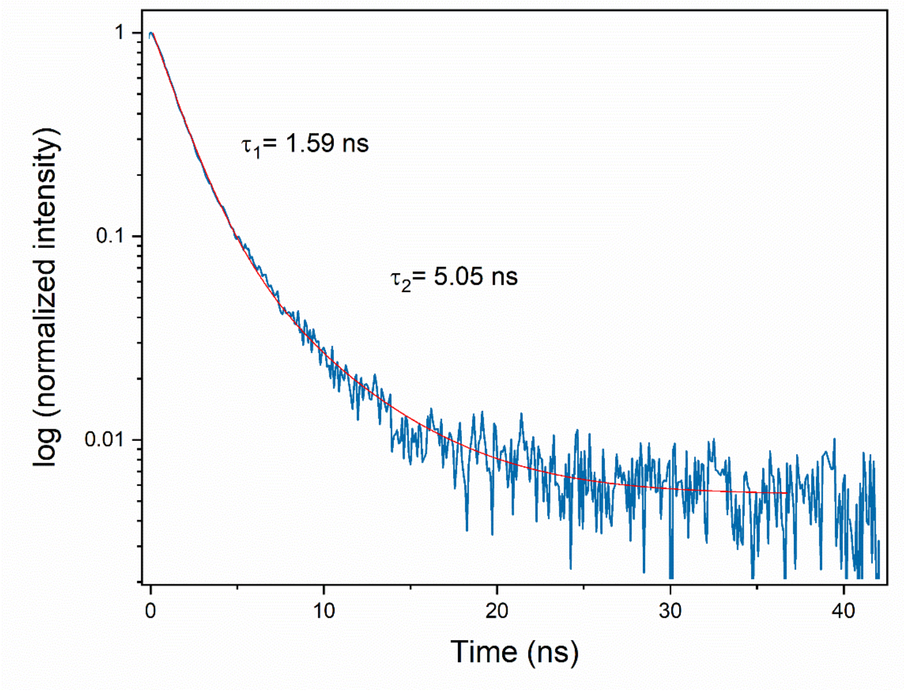

Figure S18. Emission decay curve of the CEA_2PbCl_4 sample monitored at 550 nm.

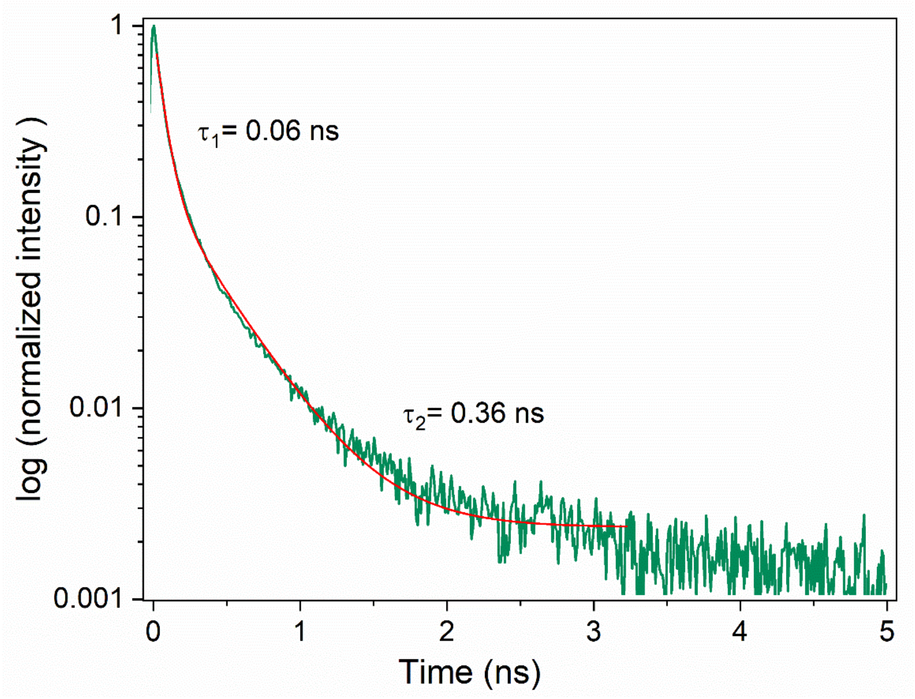

Figure S19. Emission decay curve of the CEA_2PbBr_4 sample monitored at 610 nm.

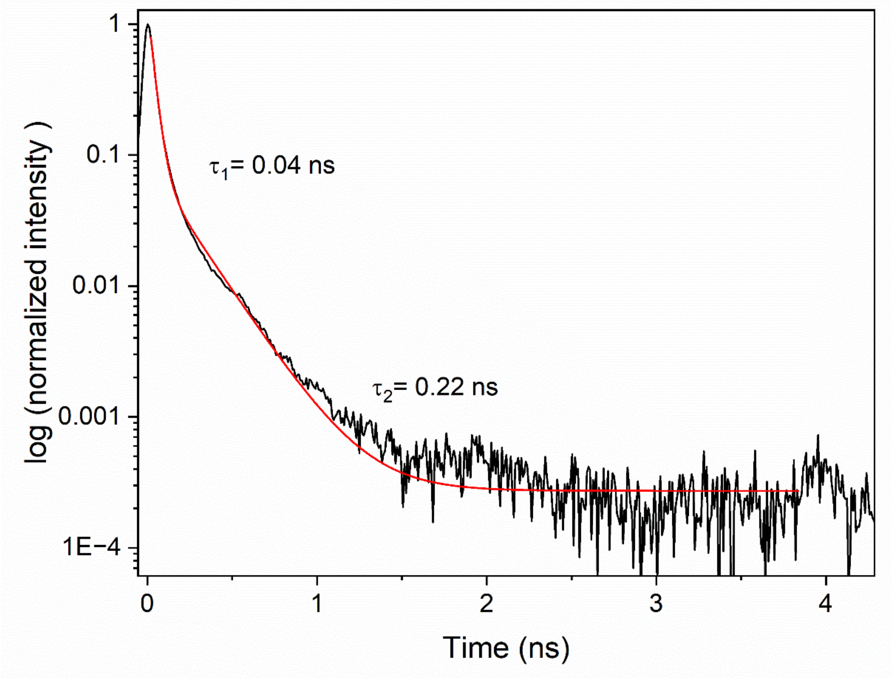

Figure S20. Emission decay curve of the CEA_2PbI_4 sample monitored at 570 nm.
